# Machine learning for lymph node metastasis prediction of in patients with gastric cancer: A systematic review and meta-analysis

**DOI:** 10.3389/fonc.2022.946038

**Published:** 2022-08-18

**Authors:** Yilin Li, Fengjiao Xie, Qin Xiong, Honglin Lei, Peimin Feng

**Affiliations:** Department of Gastroenterology, Hospital of Chengdu University of Traditional Chinese Medicine, Chengdu, China

**Keywords:** Machine learning, gastric cancer, lymph node metastasis, systematic review, meta-analysis

## Abstract

**Objective:**

To evaluate the diagnostic performance of machine learning (ML) in predicting lymph node metastasis (LNM) in patients with gastric cancer (GC) and to identify predictors applicable to the models.

**Methods:**

PubMed, EMBASE, Web of Science, and Cochrane Library were searched from inception to March 16, 2022. The pooled c-index and accuracy were used to assess the diagnostic accuracy. Subgroup analysis was performed based on ML types. Meta-analyses were performed using random-effect models. Risk of bias assessment was conducted using PROBAST tool.

**Results:**

A total of 41 studies (56182 patients) were included, and 33 of the studies divided the participants into a training set and a test set, while the rest of the studies only had a training set. The c-index of ML for LNM prediction in training set and test set was 0.837 [95%CI (0.814, 0.859)] and 0.811 [95%CI (0.785-0.838)], respectively. The pooled accuracy was 0.781 [(95%CI (0.756-0.805)] in training set and 0.753 [95%CI (0.721-0.783)] in test set. Subgroup analysis for different ML algorithms and staging of GC showed no significant difference. In contrast, in the subgroup analysis for predictors, in the training set, the model that included radiomics had better accuracy than the model with only clinical predictors (F = 3.546, p = 0.037). Additionally, cancer size, depth of cancer invasion and histological differentiation were the three most commonly used features in models built for prediction.

**Conclusion:**

ML has shown to be of excellent diagnostic performance in predicting the LNM of GC. One of the models covering radiomics and its ML algorithms showed good accuracy for the risk of LNM in GC. However, the results revealed some methodological limitations in the development process. Future studies should focus on refining and improving existing models to improve the accuracy of LNM prediction.

**Systematic Review Registration:**

https://www.crd.york.ac.uk/PROSPERO/, identifier CRD42022320752

## Background

Gastric cancer (GC) is the fifth most common malignancy and the third leading cause of cancer-associated death worldwide (1–3). Lymph node metastasis (LNM) is one of the most sensitive prognostic factors for patients with GC (4–6). Patients at different lymph node stages may require different degrees of lymphadenectomy or neoadjuvant therapy (7–10), and typically have different outcomes. Therefore, it is of necessity to accurately predict and evaluate LNM before making treatment decisions (11, 12).

Non-invasive imaging modalities, including computed tomography (CT), functional magnetic resonance imaging (fMRI), and B-ultrasonography, have been widely applied for the evaluation of lymph node status in GC patients. However, the performances of these techniques remain controversial due to their sensitivity, specificity, and accuracy (13–19). Sentinel lymph node (SLN) biopsy is an invasive approach that has also been adopted for LNM detection in GC (20), while is still in debates on its effectiveness. Kitagawa et al. (21) and Miyashiro et al. (22) applied two different SLN biopsy methods, but reached different false negative rates (7% and 46.4%, respectively). Endoscopic ultrasonography combined with fine needle aspiration can be used for local lymph node staging and LNM diagnosing, while the former fails to detect distant metastases (23). Several new molecular biomarkers have been found to be useful for predicting LNM of GC, but the application of these agents is limited due to high cost and complex technological requirements (24, 25). There are indeed multiple methods that have potential to diagnose LNM, whereas their performances are tied down by so many limitations and uncertainties, making it an urgent need to find a more applicable and effective method for the identification of LNM status.

Machine learning (ML) algorithm is a newly emerged technique that is capable of accurate raw data-processing, important data connections-analyzing, and accurate decision-making (26, 27). Compared with conventional statistical methods, ML model has higher prediction accuracy (28, 29). It is of critical application value in assisting disease-diagnosing and prognosis-predicting through processing massive and complex medical data (30, 31). Currently, ML has been increasingly applied for LNM prediction in GC patients (32–72). However, different types of ML prediction models have great differences in both included predictors and calculation methods of the models (73, 74), the results produced by different models are far from unanimous (75). More importantly, there is no systematic review and meta-analysis conducted to assess ML for LNM prediction in GC patients. Therefore, we reviewed and synthesized all the related studies published previously to evaluate the accuracy of ML models for LNM prediction in GC patients.

## Methods

This systematic review and meta-analysis was performed following the *Preferred Reporting Items for Systematic Reviews and Meta-Analyses* (PRISMA) guidelines (76). The study was registered on PROSPERO (Registration No. CRD42022320752).

### Literature retrieval strategy

PubMed, EMBASE, Web of Science, and Cochrane Library were systematically searched from inception to March 16, 2022 for all the related published articles. Search items mainly contained: “stomach neoplasms,” “machine learning,” and “lymphatic metastasis.” References of included articles were also searched manually for potential eligible studies. Detailed search procedures and strategy are presented in **Supplemental File 1**.

### Inclusion criteria

The studies were selected according to the following criteria:

(1) Patients were diagnosed with GC based on histopathological examination;
(2) Cohort study published in English, with the full-text available;
(3) Reported assessment of the performance of ML algorithm for LNM prediction;
(4) Clearly description for ML models and predictor variables used
(5) Reported the prediction performance indices of ML models and included sufficient data to infer the c-index and/or accuracy.

Studies meeting the following criteria were excluded:

(1) Limited sample size (less than 100);
(2) Letter, editorial, animal study, review, conference summary, consensus, case report, and guidelines;
(3) Research focusing on identifying or analyzing individual predictors, rather than the development and/or verification of models;
(4) Model performance measurements were not reported;
(5) Model building process or method was not described.

### Data extraction

Data extraction was processed by two reviewers independently (YL and FX). The list of extraction items was designed based on the modified version of *Checklist for Critical Appraisal and Data Extraction for Systematic Reviews of Prediction Modelling Studies* (CHARMS) (77). Discrepancies were resolved by a third reviewer (PF). The following data were extracted:

(1) study characteristics (authors, publication date, study-design, and country or region);
(2) cohort characteristics (number of participants, number of patients with positive LNM, and cancer stages);
(3) Feature selection algorithms, number and types of predictor in final model, types of ML prediction model, and model validation and application;
(4) prediction outcomes, including c-index, accuracy, sensitivity, and specificity.

### Risk of bias assessment

Risk of bias assessment and applicability of included studies were performed using the *Prediction Model Risk of Bias Assessment Tool* (PROBAST) (78), which includes four domains; participants, predictors, outcomes, and analysis. Risk of bias in each study was assessed based on the four domains, while the applicability was evaluated based on the first three domains. Each study was graded as “high risk”, “low risk”, or “unclear risk” (78). This process was conducted and cross-checked by two reviewers (YL and FX) independently. Discrepancies were settled by the third reviewer (PF).

### Statistical analysis

Data analysis was performed using R Statistical Software (version 4.1.1) with ‘matrix’, ‘metafor’ and ‘meta’ packages (79, 80). Subgroups were set based on ML algorithms. The c-index and accuracy for LNM prediction in GC patients, which were obtained from each study included, were measured with 95% confidence intervals (95% CIs) in the final analysis. For studies that did not report c-index, we calculated it *via* plotting receiver operating characteristic (ROC) curves based on reported probability distributions. The results from all included studies were pooled, and an overall estimated effect was evaluated using random-effect model which processed heterogeneity among studies (81). We used one-way ANOVA to discuss the differences in c-index and accuracy between the training group and the test group.

## Results

### Study selection

**Figure 1:**
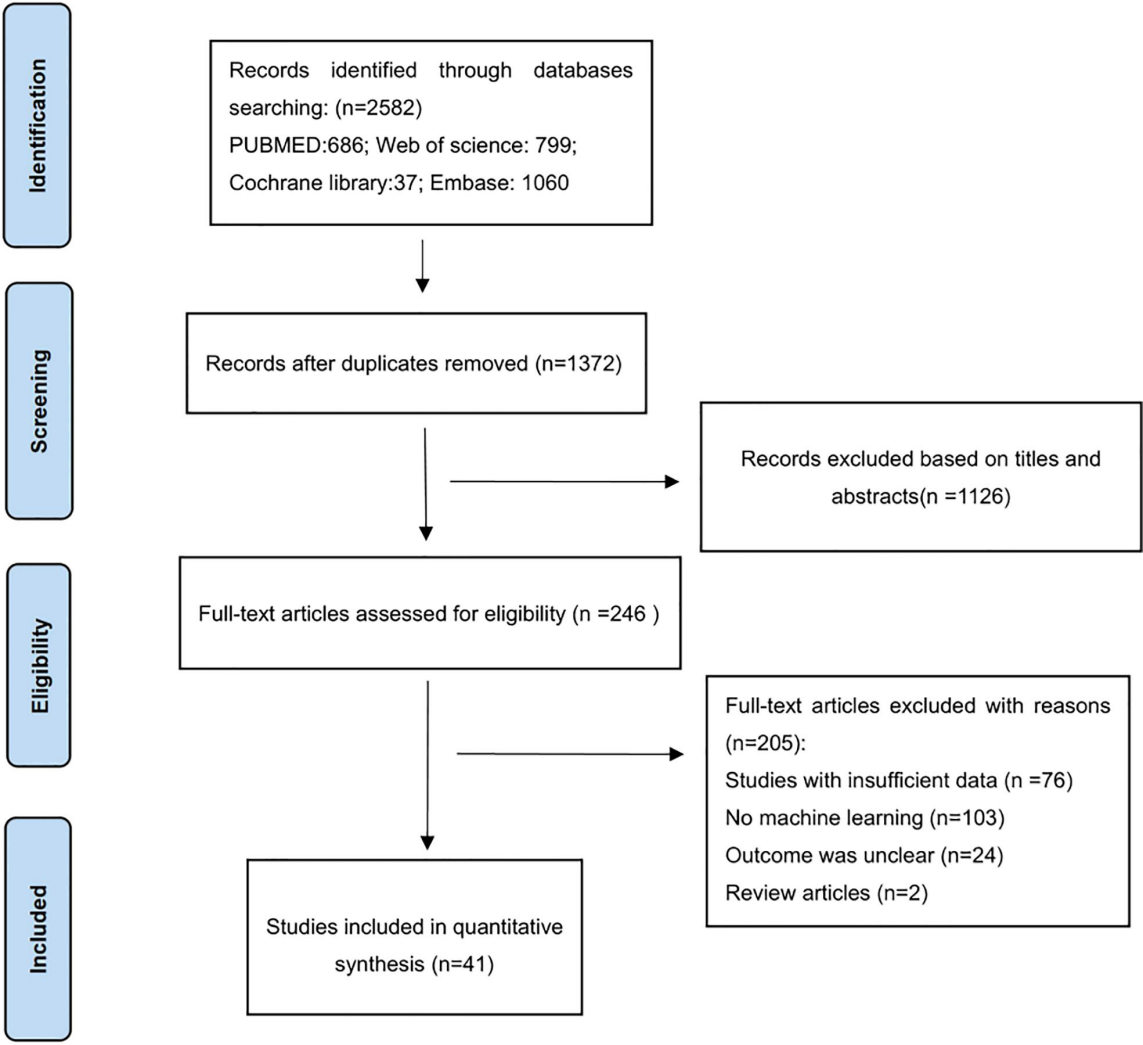
The PRISMA flow diagram for study selection.

There were 2582 articles identified through searching the four databases, in which 1210 were excluded after duplicate-checking, 1126 excluded *via* browsing titles and abstracts. Full-texts of the remaining 246 articles were read, and 205 articles were excluded for reasons specified in **Figure 1**. Finally, a total of 41 studies were included (32–72).

### Characteristics of included studies

**Figure 2:**
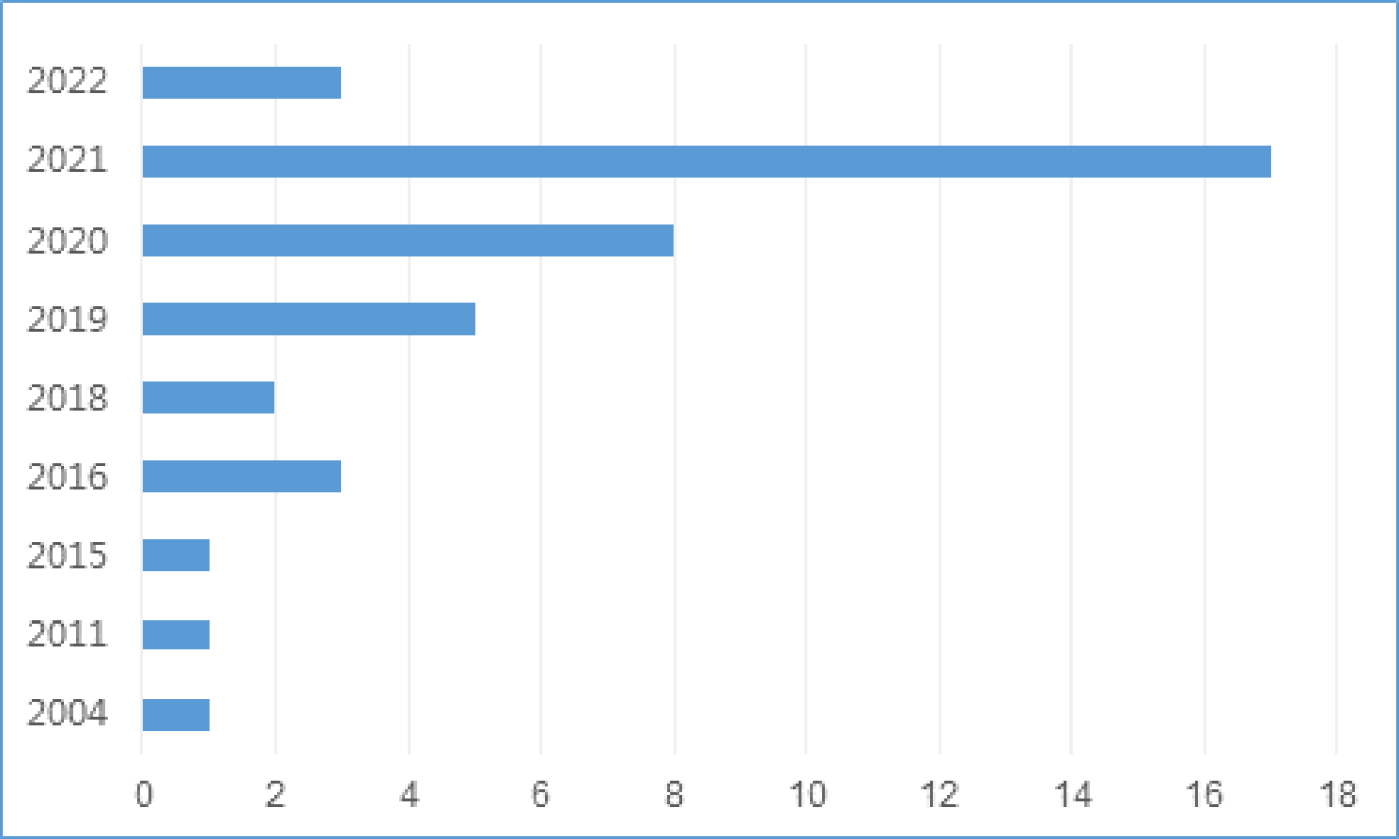
Distribution of studies by the year of publication.

There were 35 studies (85.4%) that were conducted in China (32, 33, 35–43, 45–49, 51–54, 56–63, 65–69, 71, 72), 5 (12.2%) in Korea (34, 44, 50, 55, 64) and 1 (2.4%) in Germany (70), with the publication date ranged from 2004 to 2022. The number of studies using ML for LNM prediction has gradually increased since 2018 (**Figure 2**). There were 35 retrospective studies (33, 35–53, 56–59, 61–70, 72) and 6 prospective studies (32, 34, 54, 55, 60, 71), with a total of 56182 participants, in which the number of patients with LNM was 12031. Characteristics of included studies are presented in **Table 1**.

**Table 1:** Characteristics of included studies.

Study	Country	Study design	Stage	No. patients in the train set	No. patients in the test set	Technique used for feature selection	Types of machine learning	Data source
Xiao-Peng Zhang (2011)	China	Retro	Early GC Borrmann I-IV	175	NA	LR	SVM	Single institution
Song Liu (2021)	China	Retro	Stages I-IV	122	41	LASSO	SVM, LR	Single institution
C Jin (2021)	China	Retro	Stages I-IV	1172	527	LR, RF	DL	Multiple institution
Xiaoxiao Wang (2021)	China	Retro	T1-2	80	79	LR	LR	Single institution
Xiao-Yi Yin (2020)	China	Retro	T1a, T1b	596	227	LR	LR	Single institution
Bang Wool Eom (2016)	Korea	Retro	T1a, T1b	336	NA	LR	LR	Single institution
Zhixue Zheng (2015)	China	Retro	T1a, T1b	262	NA	LR	LR	Single institution
Jing Li (2020)	China	Retro	Borrmann I-IV	136	68	LR	DL	Single institution
Zhengbing Wang (2021)	China	Retro	T1a, T1b	363	140	LR	LR	Single institution
HuaKai Tian (2022)	China	Retro	T1a, T1b	2294	227	LR	GLM, RPART, RF, GBM, SVM, RDA, ANN	Multiple institution
Zhixue Zheng (2016)	China	Retro	T1a, T1b	597	NA	LR	LR	Single institution
Yu Mei (2021)	China	Pros	T1a, T1b	794	418	LR	LR	Single institution
Jing Li (2018)	China	Retro	Borrmann I-IV	140	70	LR	LR	Single institution
Su Mi Kim (2020)	Korea	Pros	T1a, T1b	10579	2100	LR	LR	Single institution
Miaoquan Zhang (2021)	China	Retro	T1a, T1b	285	NA	LR	LR	Single institution
Yuming Jiang (2019)	China	Retro	Stages I-IV	312	1377	LR	LR	Multiple institution
Qiu-Xia Feng (2019)	China	Retro	Stages I-IV	326	164	SVM	SVM	Single institution
Jianfeng Mu (2019)	China	Retro	T1a, T1b	746	126	LR	LR	Single institution
Shilong Li (2021)	China	Retro	Stages I-IV	144	151	LR	LR	Single institution
Yue Wang (2020)	China	Retro	NA	197	50	LR	RF	Single institution
Chun Guang Guo (2016)	China	Retro	T1a, T1b	256	1273	LR	LR	Multiple institution
Cheng-Mao Zhou (2021)	China	Pros	T1a, T1b	818	351	GBDT	GBDT, XGB, RF, LR, XGB+LR, RF+LR, GBDT+LR	Single institution
Xujie Gao (2021)	China	Retro	T1a, T1b	308	155	LR	LR	Single institution
Xujie Gao (2020)	China	Retro	Stages I-IV	486	240	LR	LR	Single institution
Xu Wang (2021)	China	Retro	T1-4	250	99	LR	LR	Single institution
Siwei Pan (2021)	China	Retro	T1a, T1b	1274	637	LR	LR	Multiple institution
Wujie Chen (2019)	China	Retro	T2-4	71	75	LR	LR	Single institution
Bong-Il Song (2020)	Korea	Retro	T1-4	377	189	LR	LR	Single institution
Chao Huang (2020)	China	Retro	NA	466	NA	RF	DT	Single institution
Lili Wang (2021)	China	Retro	T2-4	340	175	LR	LR	Single institution
Seokhwi Kim (2021)	Korea	Retro	T1a	28	108	LR	Bayesian	Multiple institution
Qiufang Liu (2021)	China	Retro	NA	185	NA	RF	DL	Single institution
Wannian Sui (2021)	China	Retro	T1a, T1b	1496	246	LR	LR	Multiple institution
Dexin Chen (2019)	China	Retro	T1a, T1b	232	143	LR	LR	Multiple institution
Lingwei Meng (2021)	China	Retro	T1-4	377	162	LASSO	LR	Multiple institution
D Dong (2020)	China	Retro	T2-4	225	505	Multivariable linear regression analysis, SVM	DL	Multiple institution
Zepang Sun (2021)	China	Pros	T1-4	531	1087	LR	LR	Multiple institution
Ji-Eun Na (2022)	Korea	Pros	T1a, T1b	10332	4428	LR, SVM, RF	LR, SVM, RF	Single institution
Haixing Zhu (2022)	China	Retro	T1a, T1b	1878	470	DT, GBM, LR, ANN, RF, XGBOOST	DT, GBM, LR, ANN, RF, XGBOOST	Multiple institution
Elfriede H Bollschweiler (2004)	Germany	Retro	Stages I-IV	135	NA	ANN	ANN	Single institution
Yinan Zhang (2018)	China	Pros	T1a, T1b	272	81	LR	LR	Single institution

ANN, artificial neural network; DL, deep learning; DT, decision tree; GBM, gradient boosting machine;GC, gastric cancer; GLM, generalized linear model; LASSO, Least Absolute Shrinkage and Selection Operator; LR, logistic regression; NA, not available; No., number; Pros, prospective; RDA, regularized dual averaging; Retro, retrospective; RF, random forest; SVM, support vector machine; XGBOOST, extreme gradient boosting;

### Characteristics of machine learning in included studies

**Figure 3:**
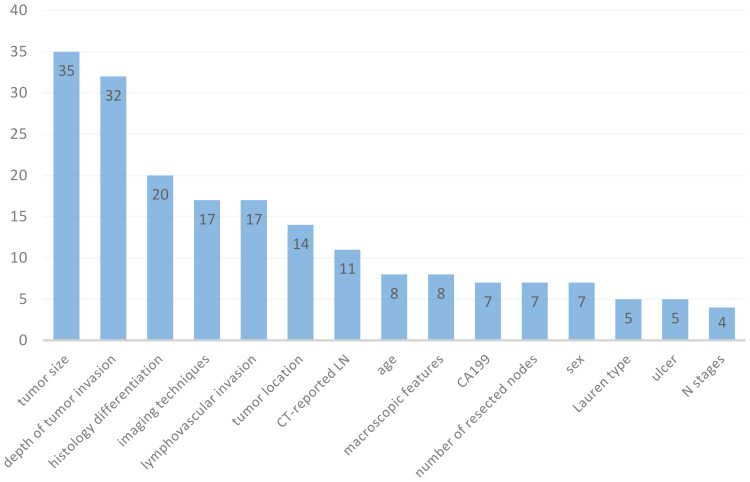
15 most frequently used predictors in 61 prediction models for gastric cancer patients.

A total of 61 models were retrieved from included studies (ranged from 1 to 7 models in each study), with various modelling methods applied. The most frequently used ML algorithms were logistic regression (LR) (n=30; 49.18%), support vector machine (SVM) (n=5; 8.2%), deep learning (DL) (n=4; 6.56%), and random forest (RF) (n=4; 6.56%) (**Table 1**). Feature selection is an important step for ML training. The number of features used in the models varied from 2 to 21, and **Figure 3** summarizes the 15 most common features. The most commonly used predictors were tumor size (n=35; 14.96%), depth of tumor invasion (n=32; 13.68%), histology differentiation (n=20; 8.55%), imaging techniques (n=17; 7.26%), lymphovascular invasion (n=17; 7.26%), tumor location (n=14; 5.98%), CT-reported LN (n=11; 4.7%), age (n=8; 3.42%), macroscopic features (n=8; 3.42%), and CA199 (n=7; 2.99%).

### Risk of bias and applicability assessment

All the included studies were of low risk of bias with respect to the domain of participants and outcome, 19 (46.34%) studies had low risk of bias in predictors (32, 34, 37, 38, 40, 42, 44, 54–56, 60, 62, 63, 66–71), 22 (53.66%) had unclear risk of bias due to that the prediction assessment was performed in the know of outcome data (33, 35, 36, 39, 41, 43, 45–53, 57–59, 61, 64, 65, 72). As for the domain of analysis, the risk of bias in 16 studies was considered high (33, 35, 41, 43, 47, 50, 51, 53, 58, 59, 61, 63, 65, 68, 70, 71), and the reasons were that (1): Insufficient sample size. Eight of the studies did not meet the standard of including at least 100 participants (2). Selection of predictors based on univariable analysis (3). Lack of external validation techniques. Eight studies lacked external validation in model development (35, 50, 51, 53, 59, 63, 65, 70). Concern regarding ‘overall applicability’ was rated as low in 15 studies (36.59%) (32, 34, 37, 38, 40, 42, 44, 54–56, 60, 62, 66, 67, 69), high in 16 studies (39.02%) (33, 35, 41, 43, 47, 50, 53, 58, 59, 61, 63, 65, 68, 70, 71) and unclear in the remaining 10 (24.39%) (36, 39, 45, 46, 48, 49, 52, 57, 64, 72). Risk of bias and applicability assessment were shown in **Table 2**.

**Table 2:** Risk of bias and applicability assessment by PROBAST criteria.

Author	Year	Risk of bias				Overall applicability rating
		Participants	Predictors	Outcome	Analysis	
Xiaoxiao Wang	2021	low	unclear	low	high	high
Xiao-Yi Yin	2020	low	unclear	low	low	unclear
Bang Wool Eom	2016	low	unclear	low	high	high
Zhixue Zheng	2015	low	unclear	low	high	high
Zhengbing Wang	2021	low	unclear	low	low	unclear
Zhixue Zheng	2016	low	unclear	low	high	high
Yu Mei	2021	low	low	low	low	low
Jing Li	2018	low	unclear	low	high	high
Su Mi Kim	2020	low	low	low	low	low
Miaoquan Zhang	2021	low	unclear	low	high	high
Yuming Jiang	2019	low	unclear	low	low	unclear
Jianfeng Mu	2019	low	low	low	low	low
Shilong Li	2021	low	low	low	low	low
Chun Guang Guo	2016	low	unclear	low	low	unclear
Xujie Gao	2021	low	unclear	low	low	unclear
Xujie Gao	2020	low	low	low	low	low
Xu Wang	2021	low	unclear	low	high	high
Siwei Pan	2021	low	low	low	low	low
Wujie Chen	2019	low	unclear	low	high	high
Bong-Il Song	2020	low	low	low	low	low
Lili Wang	2021	low	unclear	low	low	unclear
Wannian Sui	2021	low	unclear	low	low	unclear
Dexin Chen	2019	low	unclear	low	low	unclear
Zepang Sun	2021	low	low	low	low	low
Xiao-Peng Zhang	2011	low	unclear	low	high	high
Song Liu	2021	low	unclear	low	high	high
C Jin	2021	low	low	low	low	low
Jing Li	2020	low	low	low	high	high
HuaKai Tian	2022	low	low	low	low	low
Qiu-Xia Feng	2019	low	unclear	low	low	unclear
Yue Wang	2020	low	unclear	low	high	high
Cheng-Mao Zhou	2021	low	low	low	low	low
Chao Huang	2020	low	low	low	high	high
Seokhwi Kim	2021	low	unclear	low	low	unclear
Qiufang Liu	2021	low	unclear	low	high	high
Lingwei Meng	2021	low	low	low	low	low
D Dong	2020	low	low	low	low	low
Ji-Eun Na	2022	low	low	low	low	low
Haixing Zhu	2022	low	low	low	low	low
Elfriede H Bollschweiler	2004	low	low	low	high	high
Yinan Zhang	2018	low	low	low	high	high

### C-index

There were different numbers for training and test models because there were five studies which only reported the training results (35, 50–53). The overall c-index for ML in training group was 0.837 [(95%CI (0.814, 0.859)] (**Table 3**; **eFigure 1**). LR, one of the most commonly used ML methods, resulted in an overall pooled c-index of 0.838 [(95%CI (0.812, 0.865)] (**eFigure 2**), while non-logistic regression (non-LR) model resulted in an overall pooled c-index of 0.83 [(95%CI (0.786, 0.877)] (**eFigure 3**).

**Table 3:** c-index for prediction models in gastric cancer patients.

Model	Train			Test		
	No. model	c-index	95%CI	No. model	c-index	95%CI
LR	26	0.838	0.812-0.865	21	0.824	0.791-0.858
Non-LR	13	0.83	0.786-0.877	13	0.789	0.747-0.833
DL	3	0.866	0.799-0.938	3	0.835	0.780-0.895
GBDT	1	0.798	0.714-0.892	1	0.788	0.688-0.902
GBDT+LR	1	0.626	0.529-0.740	1	0.65	0.557-0.759
RF	3	0.893	0.817-0.977	3	0.848	0.829-0.868
RF+LR	1	0.691	0.594-0.804	1	0.678	0.584-0.787
SVM	2	0.847	0.804-0.894	2	0.817	0.728-0.917
XGB	1	0.881	0.786-0.987	1	0.762	0.673-0.863
XGB+LR	1	0.739	0.648-0.842	1	0.619	0.521-0.736
Overall	39	0.837	0.814-0.859	34	0.811	0.785-0.838

No. model indicates the number of prediction models. DL, deep learning; LR, logistic regression; No., number; Non-LR, non logistic regression; RF, random forest; SVM, support vector machine.

Furthermore, the pooled c-index in test group was 0.811 [(95%CI (0.785, 0.838)] (**Table 3**; **eFigure 4**), which was similar with the result in training group. Subgroup analysis showed that 21 models in LR subgroup had a pooled c-index of 0.824 [95%CI (0.791, 0.858)] (**eFigure 5**), and 13 models that used non-LR model assessment had a pooled c-index of 0.789 [95%CI (0.747, 0.833)] (**eFigure 6**).

### Accuracy

There were different numbers of training and test models because there were ten studies which only reported the results of training group (35, 50–53, 59, 63, 65, 70, 71), whereas two other studies only reported that of test group. The ML models for LNM in training group showed an overall pooled accuracy of 0.781 [95%CI (0.756-0.805)] (**Table 4**; **eFigure 7**). Subgroup analysis showed no significant difference in different ML algorithms. LR algorithms had a pooled accuracy of 0.792 [95%CI (0.761-0.82)] (**eFigure 8**) and non-LR algorithms had that of 0.768 [95%CI (0.725, 0.805)] (**eFigure 9**).

**Table 4:** Results of meta-analyses of accuracy for prediction models for gastric cancer patients.

Model	Train			Test		
	No. model	accuracy	95%CI	No. model	accuracy	95%CI
LR	28	0.792	0.761-0.820	23	0.787	0.745-0.824
Non-LR	22	0.768	0.725-0.805	18	0.707	0.665-0.746
ANN	2	0.707	0.574-0.812	1	0.634	0.589-0.678
DL	2	0.818	0.755-0.868	1	0.765	0.646-0.859
DT	1	0.794	0.754-0.830	1	0.632	0.587-0.676
GBDT	1	0.835	0.808-0.860	1	0.815	0.770-0.854
GBDT+LR	1	0.903	0.881-0.923	1	0.573	0.519-0.625
GBM	1	0.618	0.597-0.638	1	0.687	0.643-0.729
GLM	1	0.667	0.647-0.686	NA	NA	NA
RDA	1	0.668	0.649-0.688	1	0.700	0.636-0.759
RF	4	0.793	0.710-0.858	4	0.723	0.678-0.764
RF+LR	1	0.644	0.610-0.677	1	0.578	0.525-0.631
RPART	1	0.625	0.604-0.645	NA	NA	NA
SVM	4	0.765	0.678-0.835	2	0.789	0.693-0.861
XGB	1	0.863	0.838-0.886	1	0.678	0.626-0.727
XGB+LR	1	0.806	0.777-0.832	1	0.581	0.528-0.633
Bayesian	NA	NA	NA	1	0.824	0.739-0.891
XGBOOST	NA	NA	NA	1	0.691	0.648-0.733
Overall	50	0.781	0.756-0.805	41	0.753	0.721-0.783

No. model indicates the number of prediction models. ANN, artificial neural network; DL, deep learning; DT, decision tree; GBM, gradient boosting machine; GLM, generalized linear model; LR, logistic regression; NA, not available; No., number; Non-LR, non logistic regression; RDA, regularized dual averaging; RF, random forest; SVM, support vector machine; XGBOOST, extreme gradient boosting.

In test group, the accuracy of the pooled 41 models was 0.753 [95%CI (0.721-0.783)] (**Table 4**; **eFigure 10**). Subgroup analysis was conducted based on LR and non-LR algorithms assessment. The pooled accuracy for the 23 models that used LR was 0.787 [95%CI 0.745, 0.824] (**eFigure 11**). The overall pooled accuracy for non-LR models was 0.707 [95%CI (0.665, 0.746)] (**eFigure 12**).

### Subgroup analysis for early-gastric cancer and advanced gastric cancer

Of the 41 included studies, 21 were early-gastric GC (EGC) (T1) studies (32, 34, 35, 37, 39, 42, 46, 48–53, 55, 56, 60, 64, 65, 69, 71, 72) and 3 were advanced GC (T2-4) studies (43, 45, 67). In EGC, there was a pooled c-index of 0.832 [95%CI (0.804, 0.860)] (**Table 5**; **eFigure 13**) and 0.795 [95%CI (0.755, 0.838)] (**eFigure 14**) for the training and test groups, respectively. As for advanced GC, the pooled c-index for the training and test groups was 0.849 [95%CI (0.801-0.900)](**eFigure 15**) and 0.804 [95%CI (0.778-0.830)](**eFigure 16**), respectively.

**Table 5:** Subgroup analysis for early-gastric cancer and advanced gastric cancer.

Stage	Train		Test		Train		Test	
	No. model	c-index(95%CI)	No. model	c-index(95%CI)	No. model	Accuracy(95%CI)	No. model	Accuracy(95%CI)
EGC	24	0.832 (0.804-0.860)	19	0.795 (0.755-0.838)	31	0.765 (0.730-0.796)	26	0.731 (0.686-0.773)
Advanced GC	3	0.849 (0.801-0.900)	3	0.804 (0.778-0.830)	2	0.821 (0.737-0.882)	2	0.844 (0.794-0.884)

No. model indicates the number of prediction models. EGC, early-gastric cancer; GC, gastric cancer.

Thirty-one models evaluated the accuracy of ML for EGC, and their pooled accuracy was 0.765 [95% CI (0.730-0.796)](**eFigure 17**) for the training group. In terms of test group, the pooled accuracy for EGC was 0.731 [95%CI (0.686-0.773)] (**eFigure 18**). As for advanced GC, the training group had a pooled accuracy of 0.821 [95%CI (0.737-0.882)] (**eFigure 19**) while the test group had a pooled accuracy of 0.844 [95%CI (0.794-0.884)] (**eFigure 20**).

### Subgroup analysis for predictors

Furthermore, we reviewed the predictors in the included original studies and we found three cases: Group A included only clinical predictors, Group B included only radiomic predictors, and Group C included both clinical and radiomic predictors.

In the training group, the c-index of groups A, B, and C was 0.822 ± 0.079 (n = 25), 0.852 ± 0.072 (n = 8), and 0.847 ± 0.063 (n = 8), respectively, with no significant difference between them (F = 0.604, p=0.552) (**Table 6**). In the test group, the c-index of groups A, B, and C was 0.792 ± 0.092 (n = 20), 0.83 ± 0.07 (n = 8), and 0.817 ± 0.043 (n = 6), respectively, and there was also no significant difference between them (F = 0.664, p = 0.522).

**Table 6:** Subgroup analysis for predictors.

Model	Indicator	CP		RP		CP+RP		F	P
		n	mean(sd)	n	mean(sd)	n	mean(sd)		
Train	c-index	25	0.822(0.079)	8	0.852(0.072)	6	0.847(0.063)	0.604	0.552
	accuracy	34	0.75(0.087)	9	0.811(0.066)	7	0.822(0.073)	3.546	0.037
Test	c-index	20	0.792(0.092)	8	0.830(0.07)	6	0.817(0.043)	0.664	0.522
	accuracy	28	0.722(0.098)	8	0.799(0.075)	5	0.795(0.04)	3.224	0.051

n indicates the number of prediction models. CP, Clinical Predictors; RP, Radiomics Predictors.

In the training group, the accuracy of groups A, B, and C were 0.75 ± 0.087 (n=34), 0.811 ± 0.066 (n = 9), and 0.822 ± 0.073 (n = 7), respectively, and there was a significant difference between them (F = 3.546, p = 0.037) (**Table 6**), and the model containing radiomics had better accuracy. In the test group, the accuracy of groups A, B, and C were 0.722 ± 0.098 (n = 28), 0.799 ± 0.075 (n = 8), and 0.795 ± 0.04 (n = 5), respectively, although there was no significant difference between them (F = 3.224, p=0.051), the significance probability p-value was close to the critical value of 0.05. The mean value of accuracy was higher for models containing radiomics in the test cohort than for models containing only clinical predictors.

In summary, the model covering radiomics and its machine learning algorithms has better accuracy for the risk of lymph node metastasis in gastric cancer.

## Discussion

The number of studies that apply ML to LNM prediction has been gradually increasing since 2018, making it important to systematically review the published studies so as to provide guidance for future research. To our knowledge, this is the first systematic review and meta-analysis that evaluated ML performance in the assessment of LNM in GC patients. ML-related studies can be methodologically categorized into LR and non-LR study. Several included studies were assessed to be of high or unclear risk of bias in the domains of prediction, analysis and overall applicability, which highlighted the current state of technology, as well as the need for methodological quality improvement.

This study demonstrated that ML had an excellent diagnostic performance in predicting LNM with great repeatability, which was in consistence with other studies. The pooled c-index and accuracy were 0.837 [95%CI (0.814,0.859)] and 0.781 [95%CI (0.756–0.805)], respectively. Significant heterogeneity existed between the studies, which could be caused by multiple factors. EGC is defined as a tumor limited to the mucosa and submucosa, regardless of the LNM (82). A subgroup analysis was performed since the difference in the order of magnitude characteristics of LNM between EGC and advanced gastric cancer may have a certain impact on the results of machine learning. It showed no significant difference in c-index or accuracy between EGC and advanced gastric cancer. In addition, since the included studies used LR or non-LR, subgroup analysis based on this variable was conducted to observe the changes in heterogeneity between the two groups. There was also no significant difference in c-index or accuracy among different ML algorithms. The type of ML algorithm had no effect on LNM prediction. Most importantly, this study was not designed to identify one superior algorithm from the other ones.

Feature selection was also critical to the performance and interpretation of the model. The most commonly used variables in the model development were tumor size, depth of tumor invasion, histology differentiation, imaging techniques, lymphovascular invasion, tumor location, CT-reported LN, age, macroscopic features, and CA199. These variables are either anthropometric characteristics serving as markers of disease severity, or important factors contributing to the natural disease progression. These predictive indicators are easy to measure. Another merit of these predictors is the low risk of bias in measurement, resulting in a minimal possibility of exposure misclassification. Previous studies have revealed that the size of tumor is closely related to the incidence of LNM in patients with GC (83–85). Larger tumor typically indicates a higher risk of LNM (86–88), which might be attributed to easiness of invasion for larger tumor to the surrounding tissues. Depth of tumor invasion was found to be a strong predictor in 32 models (32, 34–37, 39, 41, 42, 46, 49, 50, 52, 54–56, 62, 69–71), which is in consistence with substantial evidence supporting its use as a predictor of LNM (36, 63, 89–91). There were 20 models considered histology differentiation as a vital factor for predicting LNM (34–37, 41, 42, 48–55, 61–63). Deeply infiltrated and poorly differentiated tumors might have sufficient nutritional support to facilitate its invasion to tissues, capillaries and lymphatic vessels, and thus to have the potential to grow and metastasize faster (69).

The novel PROBAST was applied for assessment of risk of bias and applicability of included prediction model studies, which allowed more details of the model, such as data source, processing, number of events per variable, feature selection, model development, and model validation, to be checked intensively (92–95). The PROBAST quality assessment revealed some other issues that could be avoided in future studies. First, external validation is rarely performed, which might be a primary limitation in studies of this field. Simple determination of samples for modeling would lead to an overestimation for the model performance (96), and further accuracy verification for these models would be advisable. Guidelines that include external validation should be followed when reporting ML models (97). On the other hand, most of the included studies were retrospective design, leading to confounding and selection bias. More prospective studies are needed to produce evidence of high quality. The included studies also demonstrated another roadblock to the clinical implementation of ML. The data used in most of the included studies were from single institution, which resulted in limited datasets for training and failed to exert the advantage of ML that it is effective in processing large samples on multiple dimensions (98). Also, limited number of studies were less likely to be of broad public health significance. Future studies should take into accounts the expansion of datasets from multiple centers to increase the sample size and to improve classifier performance.

We also note the importance of preoperative assessment of peritoneal metastasis of GC for prognosis. Currently, the assessment of peritoneal metastases is mainly in the form of radiomics, but the data obtained by radiomics is obtained from a variety of sources, usually by CT, which may affect the results obtained (99–106). The heterogeneity of the results can be brought about by the different parameters and bits of CT and the artificial partitioning by different investigators through their own experience, so that the prediction of preoperative peritoneal metastases based on radiomics can be highly heterogeneous. At the same time, the application of radiomics generates a large amount of high-dimensional data, and the screening of these high-dimensional data is a great challenge in clinical practice. Therefore, although the prediction of preoperative peritoneal metastasis based on radiomics has been favored by a large number of researchers in recent years, these studies have not reached a clear consensus, thus resulting in a great variation in C-index (C-index ranged from 0.712 to 0.981) (99–106). We also expect subsequent studies based on radiomics to guide preoperative peritoneal metastases.

There were several limitations in this study. The first limitation was the significant heterogeneity. The sample sizes and distributions varied in different studies, as well as heterogenous variety of feature selection methods and ML algorithms, which compromised the performance and applicability of each model. However, such heterogeneity could be deemed as a key finding that should be addressed by future studies. Second, the estimation for prediction performance was based on limited data due to the incomplete reports of the results in several studies. Third, most of the reviewed studies included GC patients in different cancer stages, which might represent a possible confounding factor that disrupted ML performance in differential diagnosis. Fourth, our findings should be interpreted prudently considering the potential significant publication bias. It is not suggested for investigators to report a test of unsatisfactory prediction values. It is probable that there might be instances in which ML might not have optimal prediction accuracies and so that has not been published yet (107). Last but not least, eight of the included studies that did not report the test set also affected the robustness of this study by causing a false high-performance result. It would be even better if all the studies provided external test results.

## Conclusion

ML has shown excellent diagnostic performance for LNM prediction in GC patients, and ML models based on radiomics and clinical features could be a better potential prediction method. However, there were some methodological limitations in their development, and there is still room for improvement in predictive value. Future studies are needed to explore efficient, minimally invasive, and easily collected predictors for LNM so as to build more effective ML models and improve the accuracy of LNM prediction.

## Data availability statement

The original contributions presented in the study are included in the article/**Supplementary Material**. Further inquiries can be directed to the corresponding author.

## Author contributions

Concept and design, YL, FX, QX, HL, and PF. Acquisition of data, YL, FX, and PF. Statistical analysis, YL, QX, and PF. Interpretation of data, YL, HL, and PF. Writing original draft, YL and PF. Writing review and editing, all authors. All authors have made substantial contributions to this work and have approved the final version of the manuscript.

## Funding

This work support by the National Natural Science Foundation of China. (Grant No. 81673854).

## Conflict of interest

The authors declare that the research was conducted in the absence of any commercial or financial relationships that could be construed as a potential conflict of interest.

## Publisher’s note

All claims expressed in this article are solely those of the authors and do not necessarily represent those of their affiliated organizations, or those of the publisher, the editors and the reviewers. Any product that may be evaluated in this article, or claim that may be made by its manufacturer, is not guaranteed or endorsed by the publisher.

## Supplementary material

Supplemental File 1 Search strategy

Supplementary Figure 1 The overall pooled c-index of machine learning for lymph node metastasis prediction in train set

Supplementary Figure 2 The overall pooled c-index of logistic regression models for lymph node metastasis prediction in train set

Supplementary Figure 3 The overall pooled c-index of non-logistic regression models for lymph node metastasis prediction in train set

Supplementary Figure 4 The overall pooled c-index of machine learning for lymph node metastasis prediction in test set

Supplementary Figure 5 The overall pooled c-index of logistic regression models for lymph node metastasis prediction in test set

Supplementary Figure 6 The overall pooled c-index of non-logistic regression models for lymph node metastasis prediction in test set

Supplementary Figure 7 The overall pooled accuracy of machine learning for lymph node metastasis prediction in train set

Supplementary Figure 8 The overall pooled accuracy of logistic regression models for lymph node metastasis prediction in train set

Supplementary Figure 9 The overall pooled accuracy of non-logistic regression models for lymph node metastasis prediction in train set

Supplementary Figure 10 The overall pooled accuracy of machine learning for lymph node metastasis prediction in test set

Supplementary Figure 11 The overall pooled accuracy of logistic regression models for lymph node metastasis prediction in test set

Supplementary Figure 12 The overall pooled accuracy of non-logistic regression models for lymph node metastasis prediction in test set

Supplementary Figure 13 The overall pooled c-index for predicting lymph node metastasis of early-gastric cancer in train set

Supplementary Figure 14 The overall pooled c-index for predicting lymph node metastasis of early-gastric cancer in test set

Supplementary Figure 15 The overall pooled c-index for predicting lymph node metastasis of advanced gastric cancer in train set

Supplementary Figure 16 The overall pooled c-index for predicting lymph node metastasis of advanced gastric cancer in test set

Supplementary Figure 17 The overall pooled accuracy for predicting lymph node metastasis of early-gastric cancer in train set

Supplementary Figure 18 The overall pooled accuracy for predicting lymph node metastasis of early-gastric cancer in test set

Supplementary Figure 19 The overall pooled accuracy for predicting lymph node metastasis of advanced gastric cancer in train set

Supplementary Figure 20 The overall pooled accuracy for predicting lymph node metastasis of advanced gastric cancer in test set
