# Supplementary material for: Machine learning for lymph node metastasis prediction of in patients with gastric cancer: A systematic review and meta-analysis
Source: Front Oncol. 2022 Aug 18;12:946038. doi: 10.3389/fonc.2022.946038 (PMC9433672; doi:10.3389/fonc.2022.946038)
Supplement: Supplemental File 1 — Search strategy [file DataSheet_1.docx]

**Machine learning for lymph node metastasis prediction of in patients with gastric cancer: a systematic review and meta-analysis**

**Supplemental File 1** Search strategy

**Supplemental Figures**

**eFigure 1** The overall pooled c-index of machine learning for lymph node metastasis prediction in train set

**eFigure 2** The overall pooled c-index of logistic regression models for lymph node metastasis prediction in train set

**eFigure 3** The overall pooled c-index of non-logistic regression models for lymph node metastasis prediction in train set

**eFigure 4** The overall pooled c-index of machine learning for lymph node metastasis prediction in test set

**eFigure 5** The overall pooled c-index of logistic regression models for lymph node metastasis prediction in test set

**eFigure 6** The overall pooled c-index of non-logistic regression models for lymph node metastasis prediction in test set

**eFigure 7** The overall pooled accuracy of machine learning for lymph node metastasis prediction in train set

**eFigure 8** The overall pooled accuracy of logistic regression models for lymph node metastasis prediction in train set

**eFigure 9** The overall pooled accuracy of non-logistic regression models for lymph node metastasis prediction in train set

**eFigure 10** The overall pooled accuracy of machine learning for lymph node metastasis prediction in test set

**eFigure 11** The overall pooled accuracy of logistic regression models for lymph node metastasis prediction in test set

**eFigure 12** The overall pooled accuracy of non-logistic regression models for lymph node metastasis prediction in test set

**eFigure 13** The overall pooled c-index for predicting lymph node metastasis of early-gastric cancer in train set

**eFigure 14** The overall pooled c-index for predicting lymph node metastasis of early-gastric cancer in test set

**eFigure 15** The overall pooled c-index for predicting lymph node metastasis of advanced gastric cancer in train set

**eFigure 16** The overall pooled c-index for predicting lymph node metastasis of advanced gastric cancer in test set

**eFigure 17** The overall pooled accuracy for predicting lymph node metastasis of early-gastric cancer in train set

**eFigure 18** The overall pooled accuracy for predicting lymph node metastasis of early-gastric cancer in test set

**eFigure 19** The overall pooled accuracy for predicting lymph node metastasis of advanced gastric cancer in train set

**eFigure 20** The overall pooled accuracy for predicting lymph node metastasis of advanced gastric cancer in test set

**Supplemental File 1. Search strategy**

Search date: March 16, 2022

Databases searched: PubMed, EMBASE, Web of Science, and Cochrane Library

**Search Strategy in PUBMED**

(((((((((((((((((((((Stomach Neoplasms[Title/Abstract]) OR (Neoplasm, Stomach[Title/Abstract])) OR (Stomach Neoplasm[Title/Abstract])) OR (Neoplasms, Stomach[Title/Abstract])) OR (Gastric Neoplasms[Title/Abstract])) OR (Gastric Neoplasm[Title/Abstract])) OR (Neoplasm, Gastric[Title/Abstract])) OR (Neoplasms, Gastric[Title/Abstract])) OR (Cancer of Stomach[Title/Abstract])) OR (Stomach Cancers[Title/Abstract])) OR (Gastric Cancer[Title/Abstract])) OR (Cancer, Gastric[Title/Abstract])) OR (Cancers, Gastric[Title/Abstract])) OR (Gastric Cancers[Title/Abstract])) OR (Stomach Cancer[Title/Abstract])) OR (Cancer, Stomach[Title/Abstract])) OR (Cancers, Stomach[Title/Abstract])) OR (Cancer of the Stomach[Title/Abstract])) OR (Gastric Cancer, Familial Diffuse[Title/Abstract])) OR ("Stomach Neoplasms"[Mesh])) AND (("Machine Learning"[Mesh]) OR ((((((((((((((((((machine learning[Title/Abstract]) OR (Deep learning[Title/Abstract])) OR (artificial intelligence[Title/Abstract])) OR (Prediction model[Title/Abstract])) OR (Transfer Learning[Title/Abstract])) OR (random forest[Title/Abstract])) OR (artificial neural network[Title/Abstract])) OR (ANN[Title/Abstract])) OR (Support vector machine[Title/Abstract])) OR (SVM[Title/Abstract])) OR (Nomogram[Title/Abstract])) OR (XGboost[Title/Abstract])) OR (Logistic[Title/Abstract])) OR (Decision tree[Title/Abstract])) OR (c-index[Title/Abstract])) OR (ROC[Title/Abstract])) OR (AUC[Title/Abstract])) OR (External validation[Title/Abstract])))) AND ((((((Lymphatic Metastasis[Title/Abstract]) OR (Lymphatic Metastases[Title/Abstract])) OR (Lymph Node Metastasis[Title/Abstract])) OR (Lymph Node Metastases[Title/Abstract])) OR (Metastasis, Lymph Node[Title/Abstract])) OR ("Lymphatic Metastasis"[Mesh]))

**686 Results**

**Search Strategy in Embase**

| Number | String | Results |
| --- | --- | --- |
| #1 | 'stomach tumor'/exp | 181113 |
| #2 | 'stomach tumor':ab,ti OR 'stomach neoplasms':ab,ti OR 'neoplasm, stomach':ab,ti OR 'stomach neoplasm':ab,ti OR 'neoplasms, stomach':ab,ti OR 'gastric neoplasms':ab,ti OR 'gastric neoplasm':ab,ti OR 'neoplasm, gastric':ab,ti OR 'neoplasms, gastric':ab,ti OR 'cancer of stomach':ab,ti OR 'stomach cancers':ab,ti OR 'gastric cancer':ab,ti OR 'cancer, gastric':ab,ti OR 'cancers, gastric':ab,ti OR 'gastric cancers':ab,ti OR 'stomach cancer':ab,ti OR 'cancer, stomach':ab,ti OR 'cancers, stomach':ab,ti OR 'cancer of the stomach':ab,ti OR 'gastric cancer, familial diffuse':ab,ti | 110038 |
| #3 | #1 OR #2 | 192918 |
| #4 | 'machine learning'/exp | 298236 |
| #5 | 'machine learning':ab,ti OR 'deep learning':ab,ti OR 'artificial intelligence':ab,ti OR 'prediction model':ab,ti OR 'transfer learning':ab,ti OR 'random forest':ab,ti OR 'artificial neural network':ab,ti OR ann:ab,ti OR 'support vector machine':ab,ti OR svm:ab,ti OR nomogram:ab,ti OR xgboost:ab,ti OR logistic:ab,ti OR 'decision tree':ab,ti OR 'c index':ab,ti OR roc:ab,ti OR auc:ab,ti OR 'external validation':ab,ti | 970992 |
| #6 | #4 OR #5 | 1154601 |
| #7 | 'lymph node metastasis'/exp | 160208 |
| #8 | 'lymph node metastasis':ab,ti OR 'lymphatic metastasis':ab,ti OR 'lymphatic metastases':ab,ti OR 'lymph node metastases':ab,ti OR 'metastasis, lymph node':ab,ti | 79407 |
| #9 | #7 OR #8 | 174120 |
| #10 | #3 AND #6 AND #9 | 1060 |

**1060 Results**

**Search Strategy in Web of science**

| Number | String | Results |
| --- | --- | --- |
| #1 | Stomach Neoplasms (Topic) or Neoplasm, Stomach (Topic) or Stomach Neoplasm (Topic) or Neoplasms, Stomach (Topic) or Gastric Neoplasms (Topic) or Gastric Neoplasm (Topic) or Neoplasm, Gastric (Topic) or Neoplasms, Gastric (Topic) or Cancer of Stomach (Topic) or Stomach Cancers (Topic) or Gastric Cancer (Topic) or Cancer, Gastric (Topic) or Cancers, Gastric (Topic) or Gastric Cancers (Topic) or Stomach Cancer (Topic) or Cancer, Stomach (Topic) or Cancers, Stomach (Topic) or Cancer of the Stomach (Topic) or Gastric Cancer, Familial Diffuse (Topic) or Stomach tumor (Topic) | 134910 |
| #2 | machine learning (Topic) or Deep learning (Topic) or artificial intelligence (Topic) or Prediction model (Topic) or Transfer Learning (Topic) or random forest (Topic) or artificial neural network (Topic) or ANN (Topic) or Support vector machine (Topic) or SVM (Topic) or Nomogram (Topic) or XGboost (Topic) or Logistic (Topic) or Decision tree (Topic) or c-index (Topic) or ROC (Topic) or AUC (Topic) or External validation (Topic) | 2030409 |
| #3 | Lymphatic Metastasis (Topic) or Lymphatic Metastases (Topic) or Lymph Node Metastasis (Topic) or Lymph Node Metastases (Topic) or Metastasis, Lymph Node (Topic) | 95415 |
| #4 | #1 AND #2 AND #3 | 799 |

**799 Results**

**Search Strategy in the Cochrane CENTRAL Library**

| Number | String | Results |
| --- | --- | --- |
| #1 | MeSH descriptor: [Stomach Neoplasms] explode all trees | 2808 |
| #2 | (Stomach Neoplasms):ti,ab,kw OR (Neoplasm, Stomach):ti,ab,kw OR (Stomach Neoplasm):ti,ab,kw OR (Neoplasms, Stomach):ti,ab,kw OR (Gastric Neoplasms):ti,ab,kw | 4301 |
| #3 | (Gastric Neoplasm):ti,ab,kw OR (Neoplasm, Gastric):ti,ab,kw OR (Neoplasms, Gastric):ti,ab,kw OR (Cancer of Stomach):ti,ab,kw OR (Stomach Cancers):ti,ab,kw | 7558 |
| #4 | (Gastric Cancer):ti,ab,kw OR (Cancer, Gastric):ti,ab,kw OR (Cancers, Gastric):ti,ab,kw OR (Gastric Cancers):ti,ab,kw OR (Stomach Cancer):ti,ab,kw | 9446 |
| #5 | (Cancer, Stomach):ti,ab,kw OR (Cancers, Stomach):ti,ab,kw OR (Cancer of the Stomach):ti,ab,kw OR (Gastric Cancer, Familial Diffuse):ti,ab,kw OR (Stomach tumor):ti,ab,kw | 7049 |
| #6 | #1 or #2 or #3 or #4 or #5 | 10561 |
| #7 | MeSH descriptor: [Machine Learning] explode all trees | 200 |
| #8 | (machine learning):ti,ab,kw OR (Deep learning):ti,ab,kw OR (artificial intelligence):ti,ab,kw OR (Prediction model):ti,ab,kw OR (Transfer Learning):ti,ab,kw | 8872 |
| #9 | (random forest):ti,ab,kw OR (artificial neural network):ti,ab,kw OR (ANN):ti,ab,kw OR (Support vector machine):ti,ab,kw OR (SVM):ti,ab,kw | 3023 |
| #10 | (Nomogram):ti,ab,kw OR (XGboost):ti,ab,kw OR (Logistic):ti,ab,kw OR (Decision tree):ti,ab,kw OR (c-index):ti,ab,kw | 29298 |
| #11 | (ROC):ti,ab,kw OR (AUC):ti,ab,kw OR (External validation):ti,ab,kw | 24634 |
| #12 | #7 or #8 or #9 or #10 or #11 | 60449 |
| #13 | MeSH descriptor: [Lymphatic Metastasis] explode all trees | 1901 |
| #14 | (Lymphatic Metastasis):ti,ab,kw OR (Lymphatic Metastases):ti,ab,kw OR (Lymph Node Metastasis):ti,ab,kw OR (Lymph Node Metastases):ti,ab,kw OR (Metastasis, Lymph Node):ti,ab,kw | 5756 |
| #15 | #13 or #14 | 5756 |
| #16 | #6 and #12 and #15 | 37 |

**37 Results**


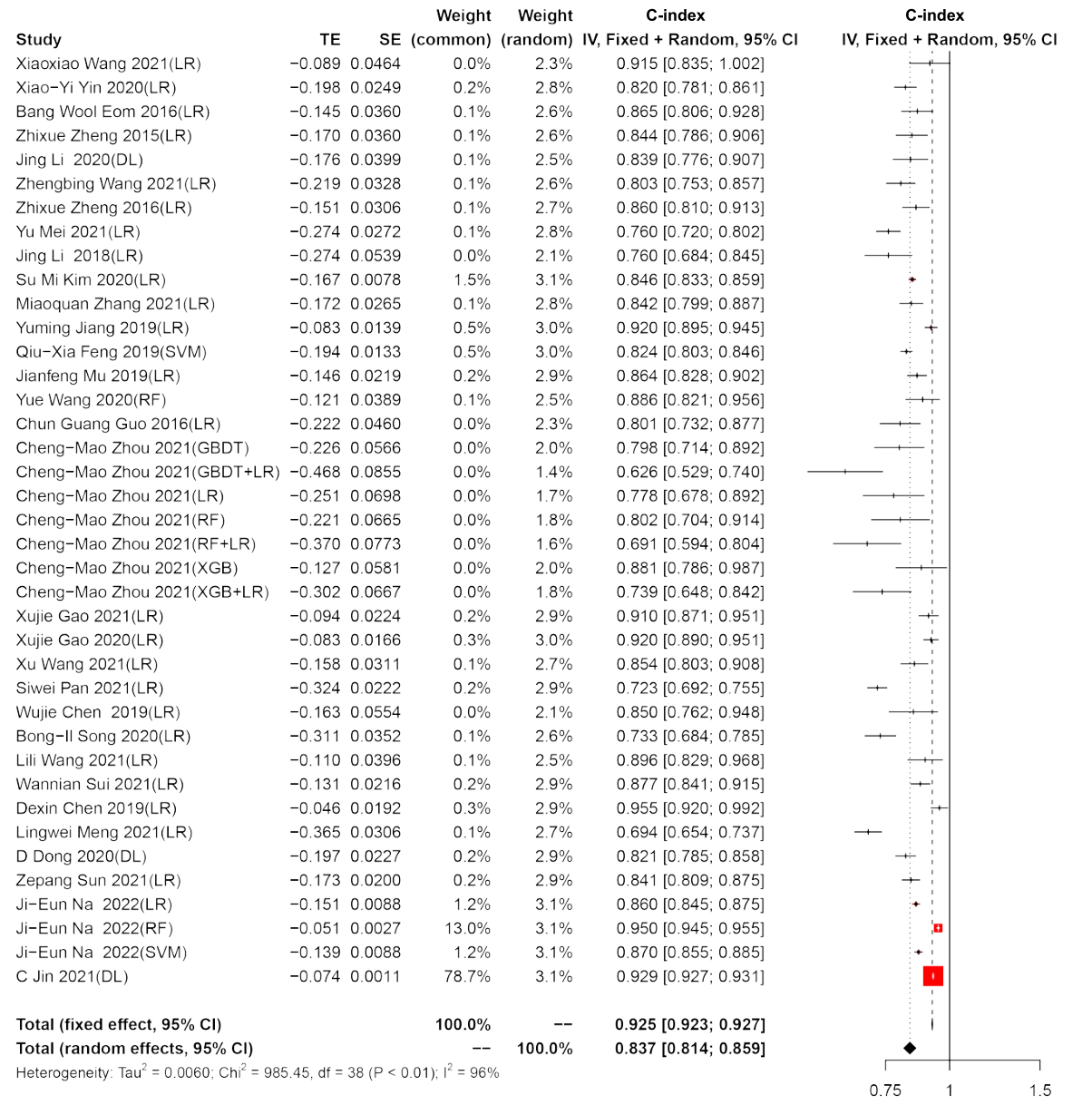


**eFigure 1** The overall pooled c-index of machine learning for lymph node metastasis prediction in train set


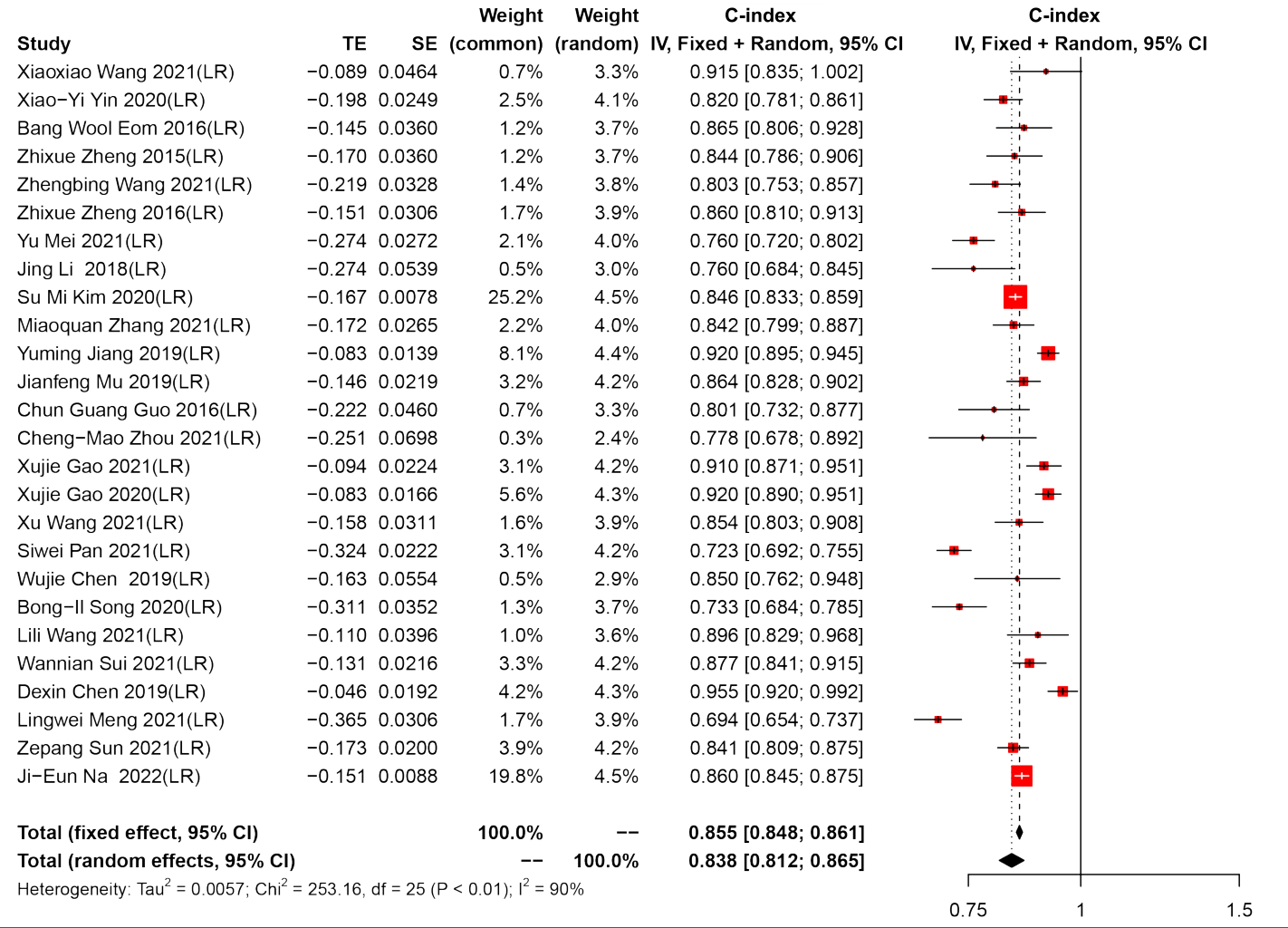


**eFigure 2** The overall pooled c-index of logistic regression models for lymph node metastasis prediction in train set


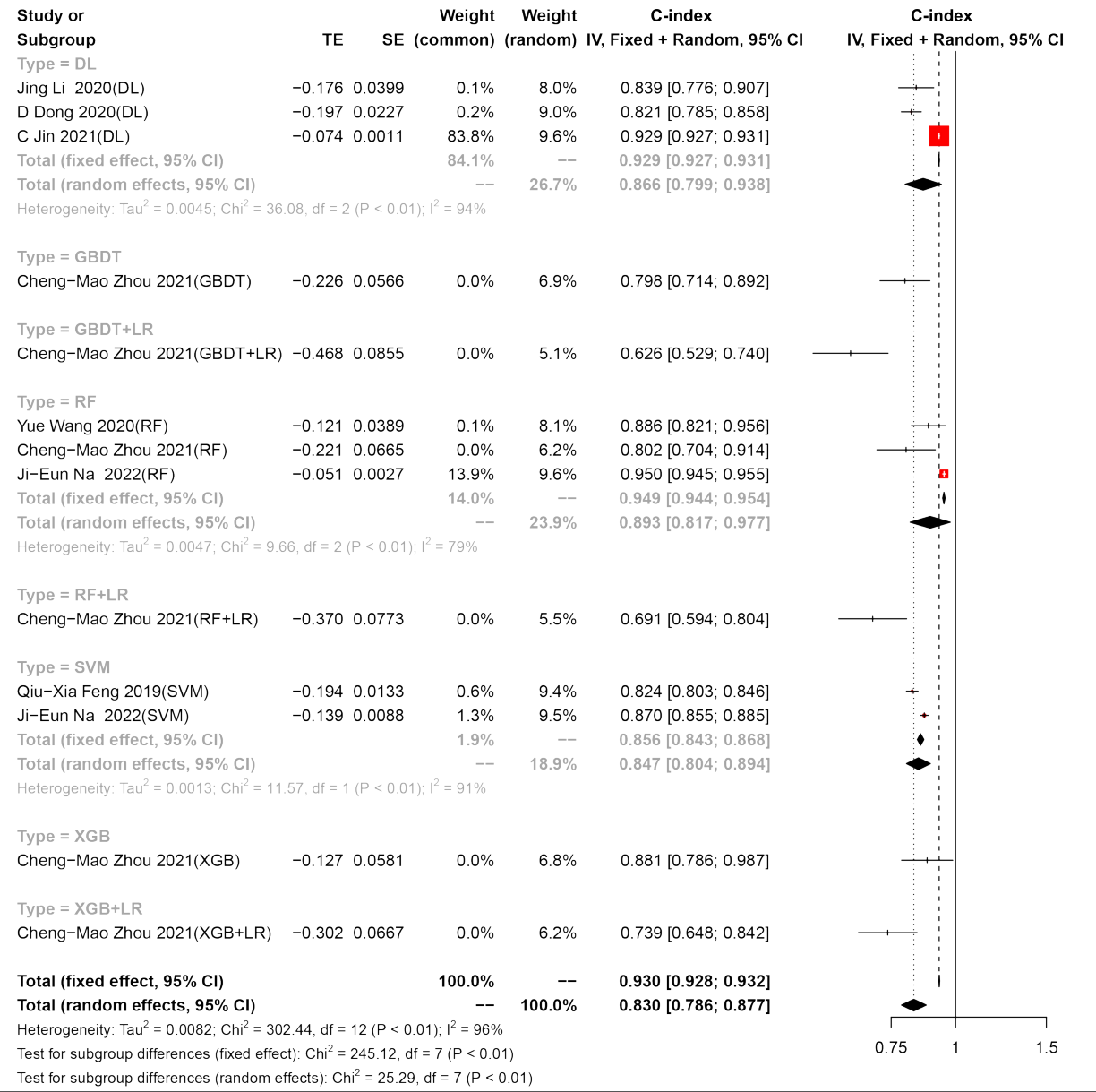


**eFigure 3** The overall pooled c-index of non-logistic regression models for lymph node metastasis prediction in train set


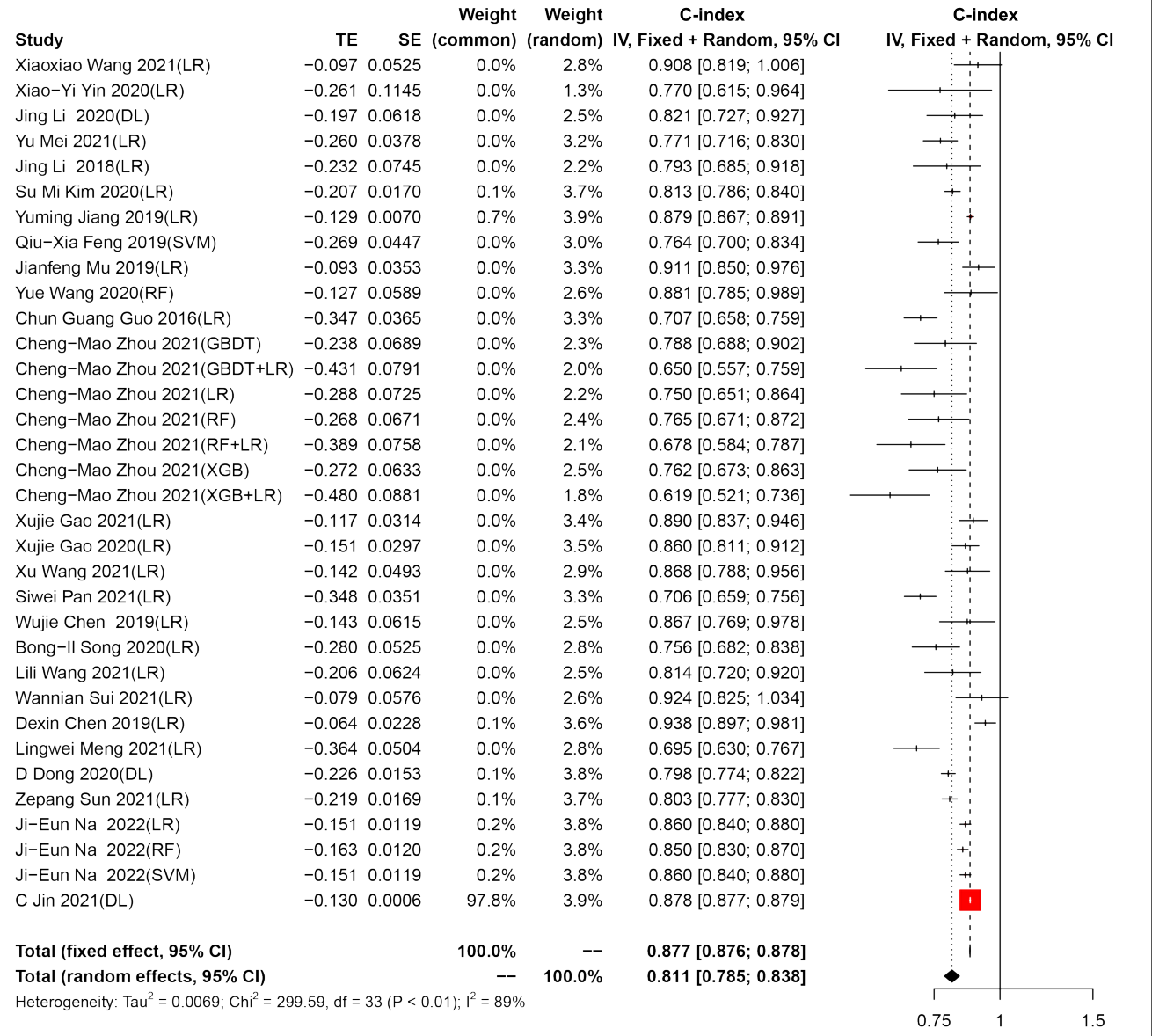


**eFigure 4** The overall pooled c-index of machine learning for lymph node metastasis prediction in test set


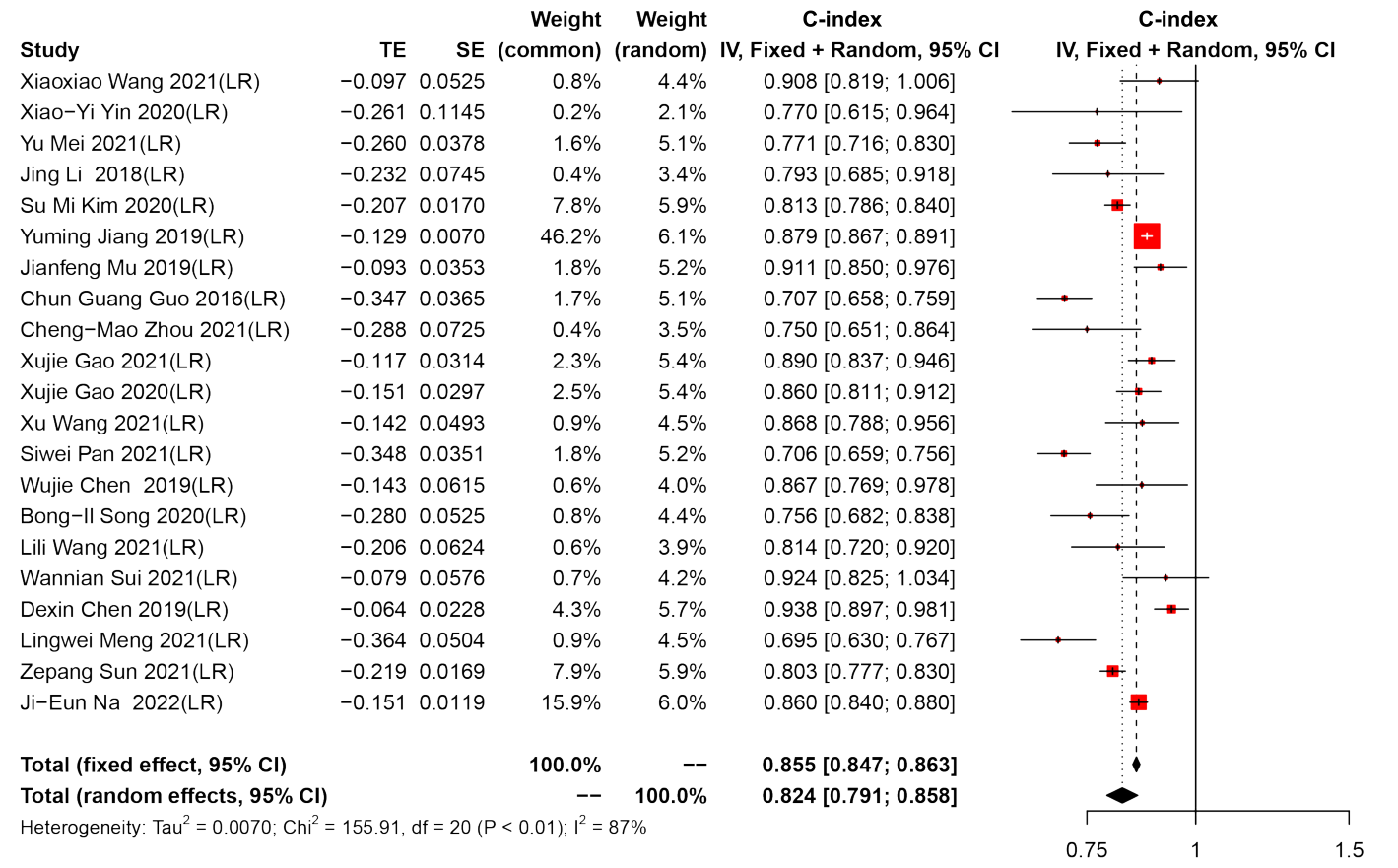


**eFigure 5** The overall pooled c-index of logistic regression models for lymph node metastasis prediction in test set


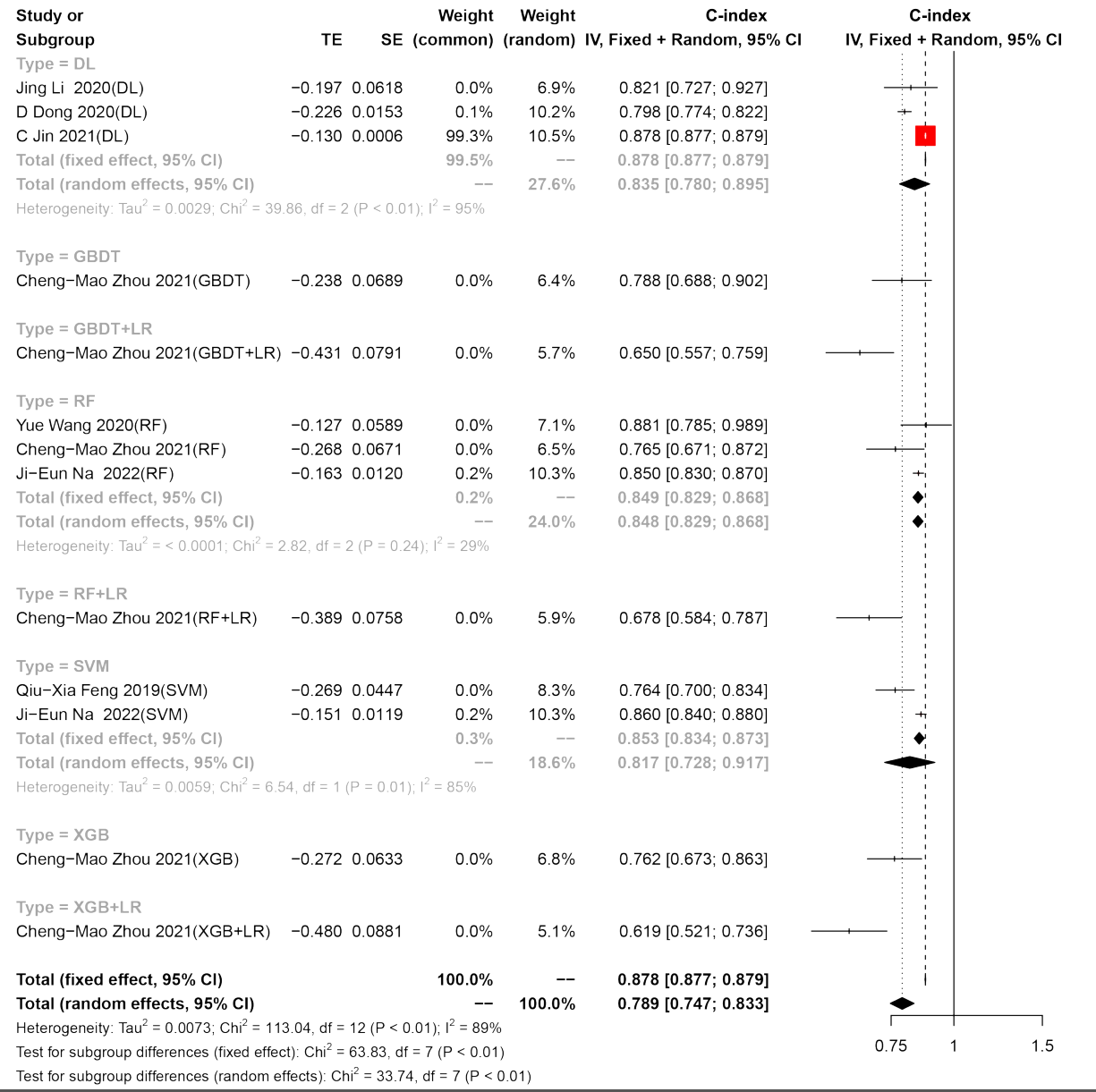


**eFigure 6** The overall pooled c-index of non-logistic regression models for lymph node metastasis prediction in test set


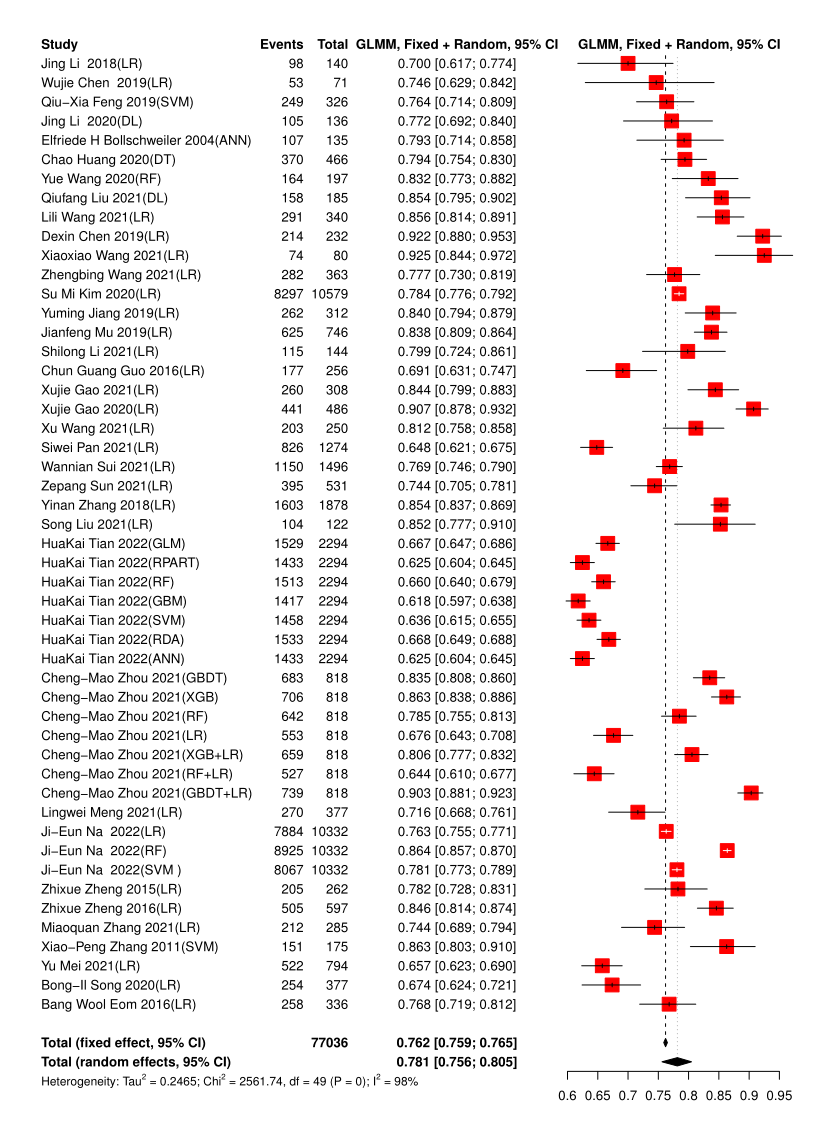


**eFigure 7** The overall pooled accuracy of machine learning for lymph node metastasis prediction in train set


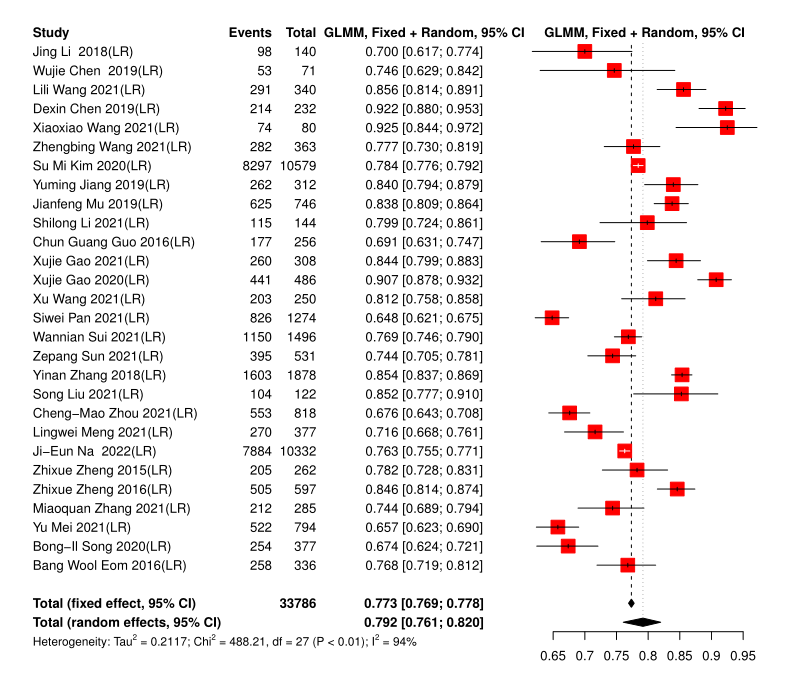


**eFigure 8** The overall pooled accuracy of logistic regression models for lymph node metastasis prediction in train set


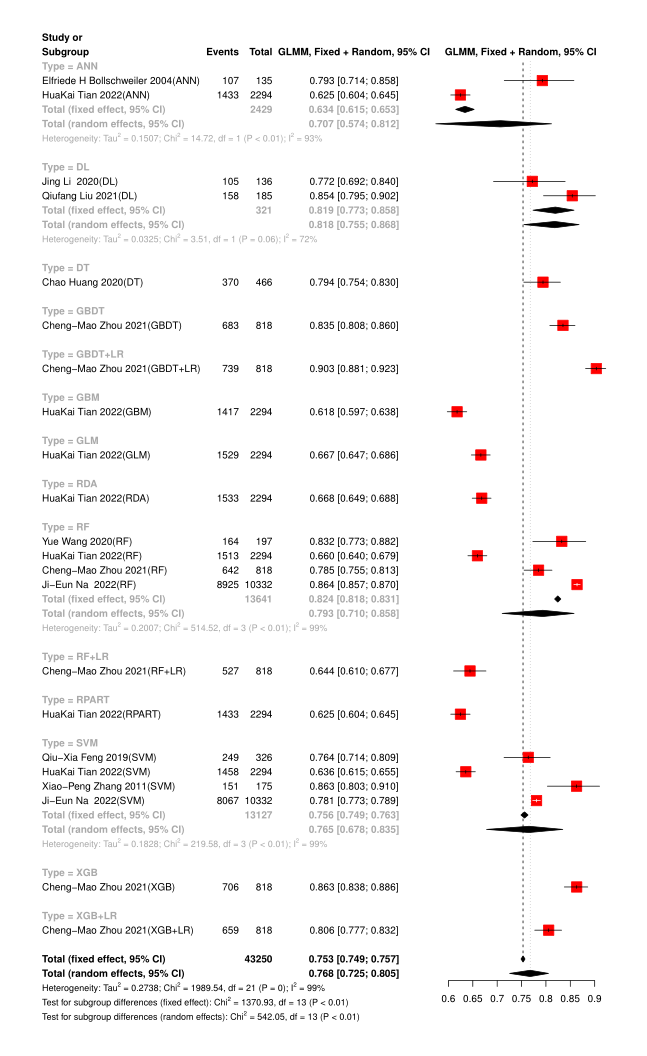


**eFigure 9** The overall pooled accuracy of non-logistic regression models for lymph node metastasis prediction in train set


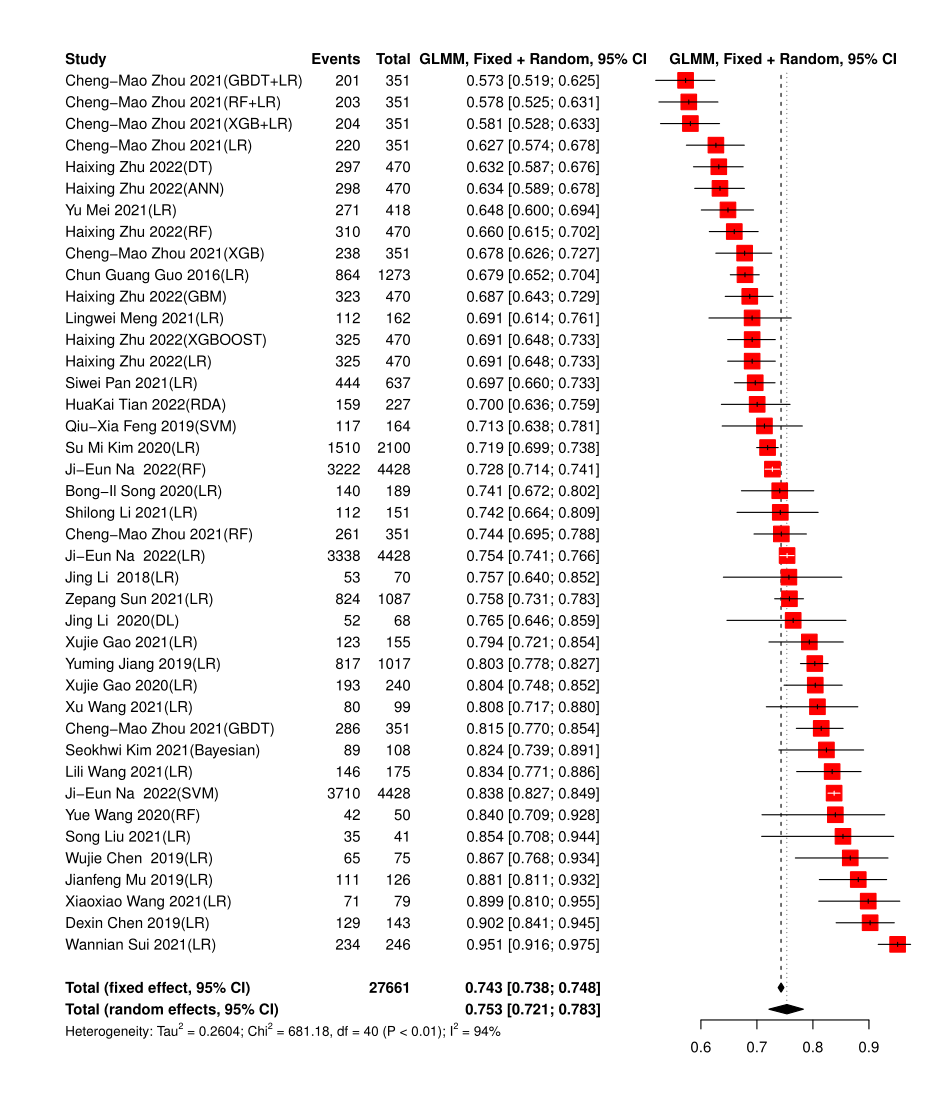


**eFigure 10** The overall pooled accuracy of machine learning for lymph node metastasis prediction in test set


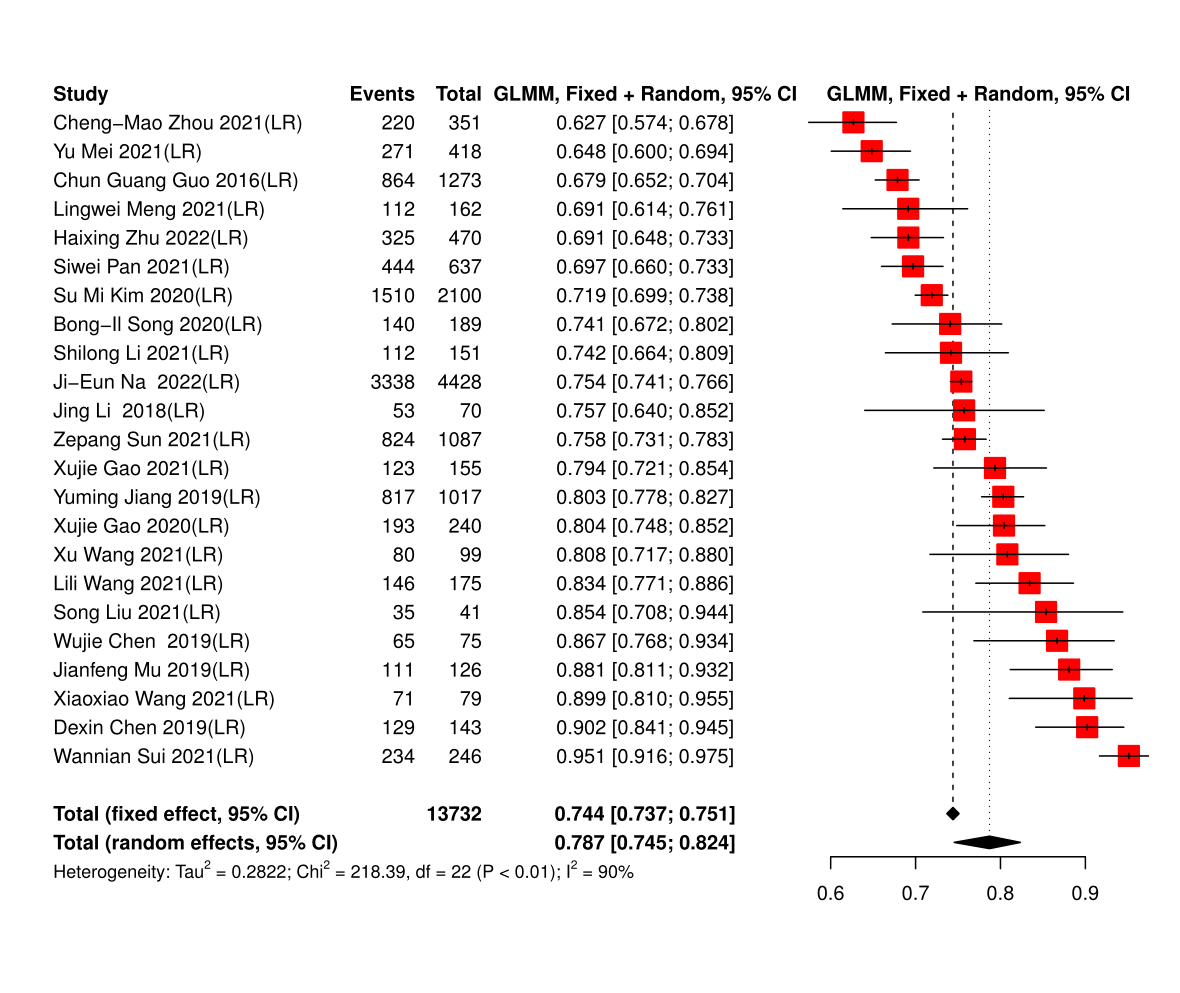


**eFigure 11** The overall pooled accuracy of logistic regression models for lymph node metastasis prediction in test set


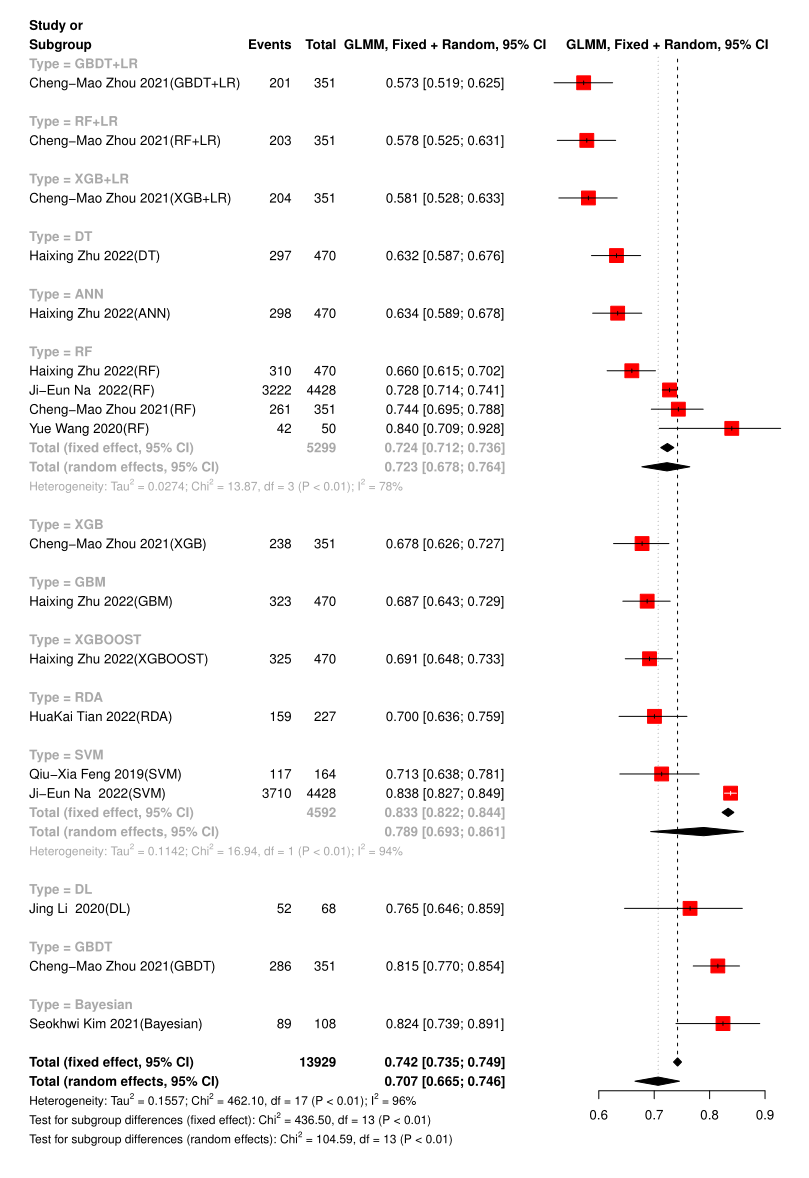


**eFigure 12** The overall pooled accuracy of non-logistic regression models for lymph node metastasis prediction in test set


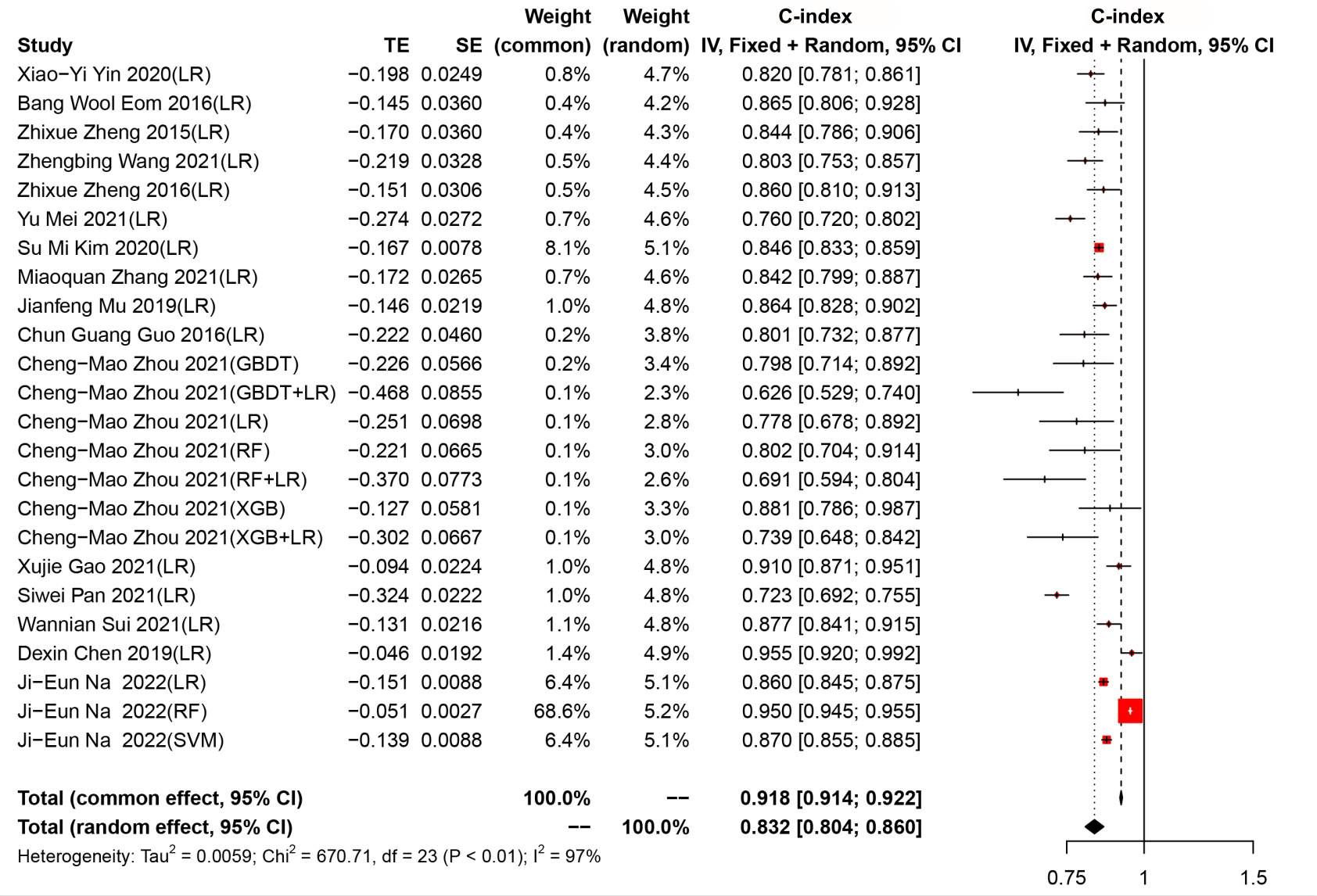


**eFigure 13** The overall pooled c-index for predicting lymph node metastasis of early-gastric cancer in train set


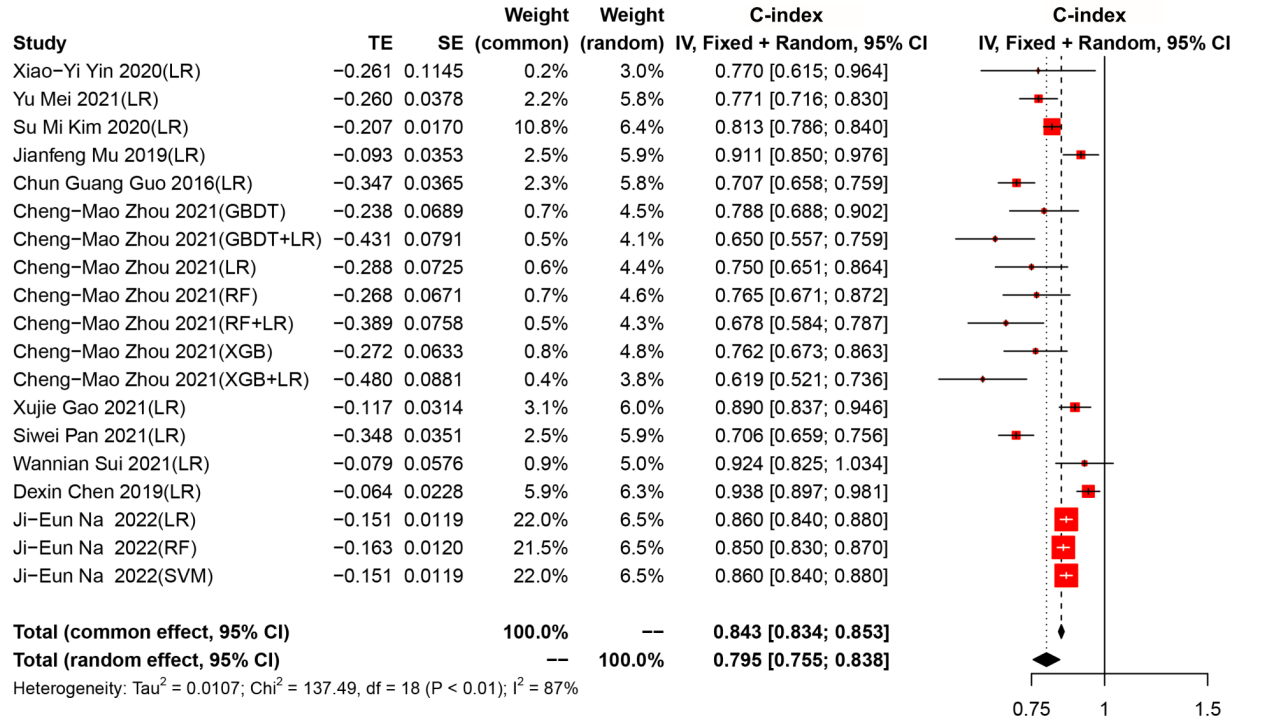


**eFigure 14** The overall pooled c-index for predicting lymph node metastasis of early-gastric cancer in test set


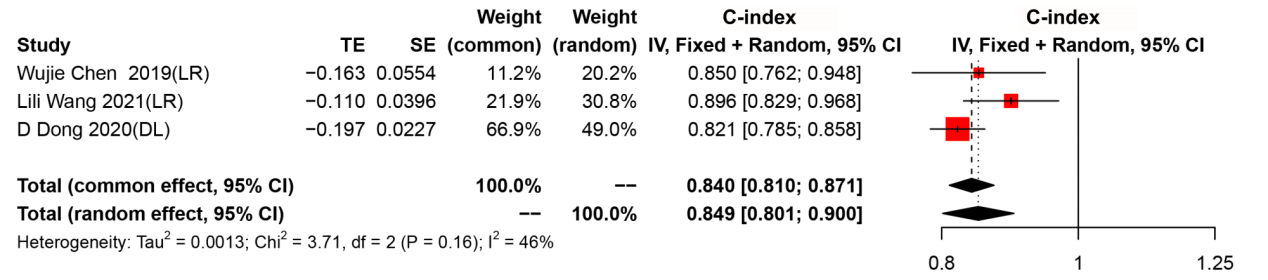


**eFigure 15** The overall pooled c-index for predicting lymph node metastasis of advanced gastric cancer in train set


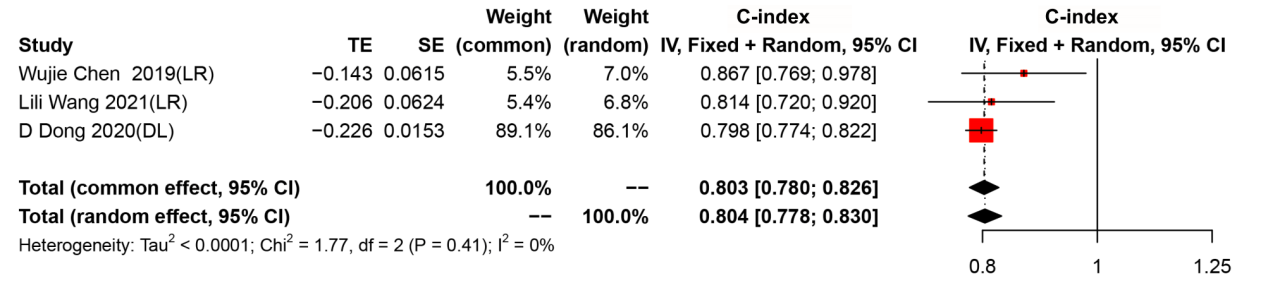


**eFigure 16** The overall pooled c-index for predicting lymph node metastasis of advanced gastric cancer in test set


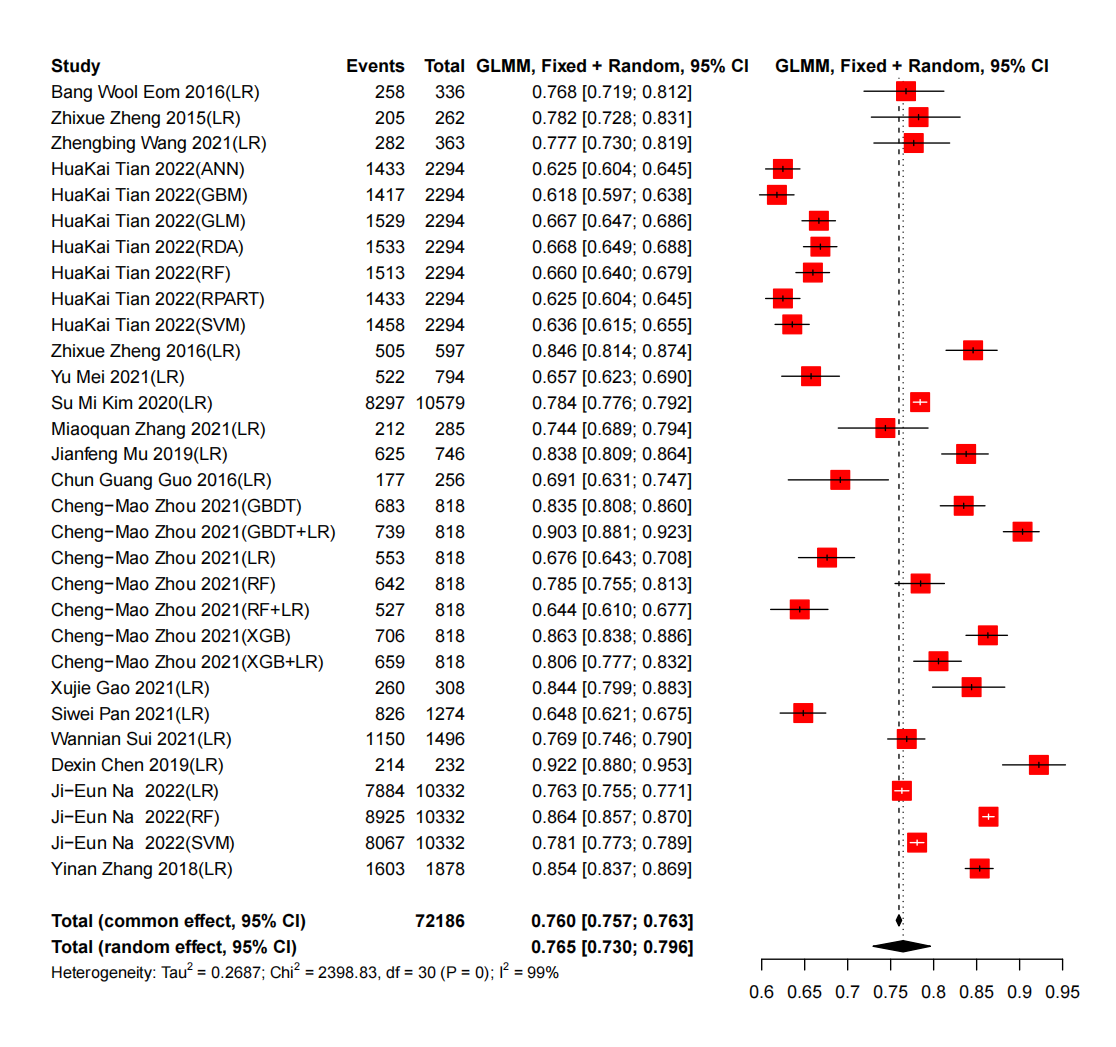


**eFigure 17** The overall pooled accuracy for predicting lymph node metastasis of early-gastric cancer in train set


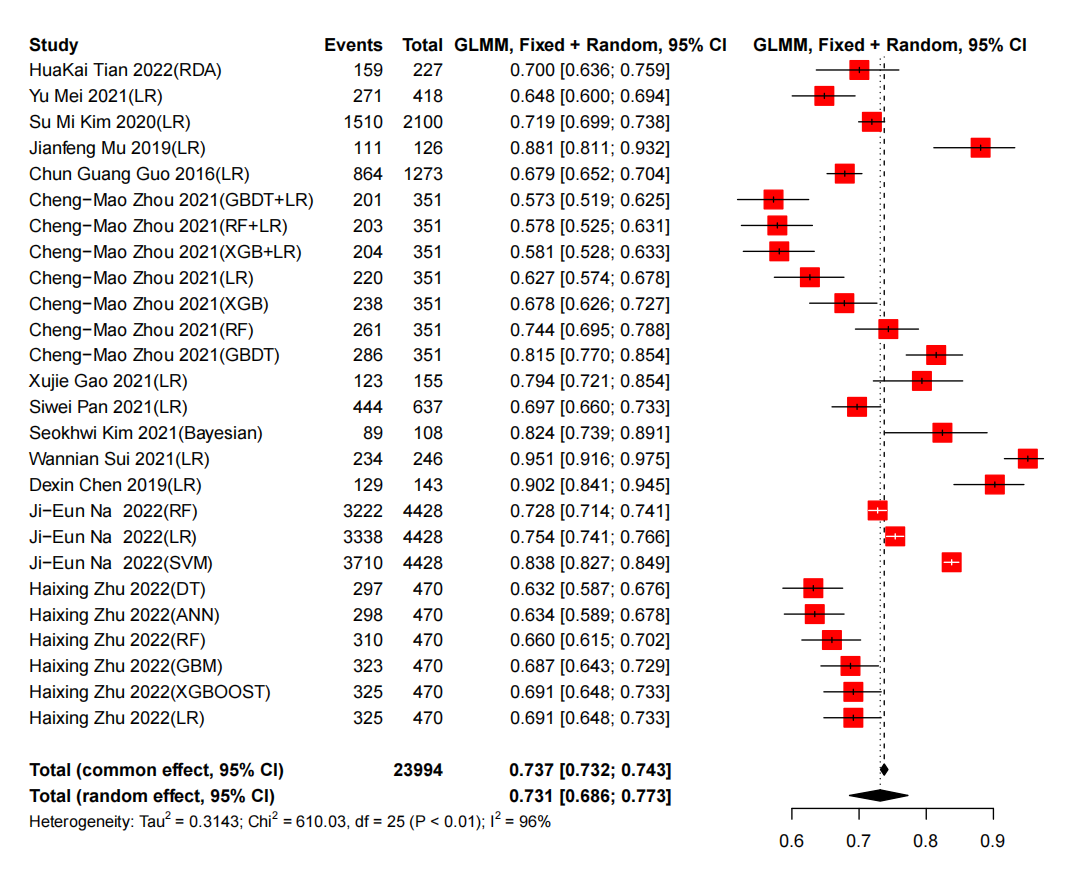


**eFigure 18** The overall pooled accuracy for predicting lymph node metastasis of early-gastric cancer in test set


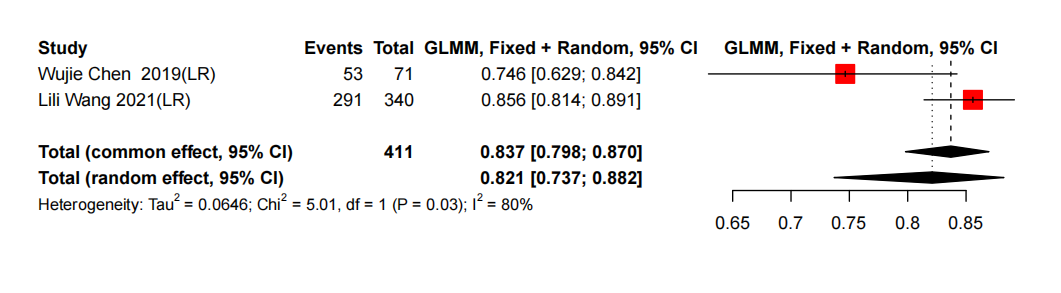


**eFigure 19** The overall pooled accuracy for predicting lymph node metastasis of advanced gastric cancer in train set


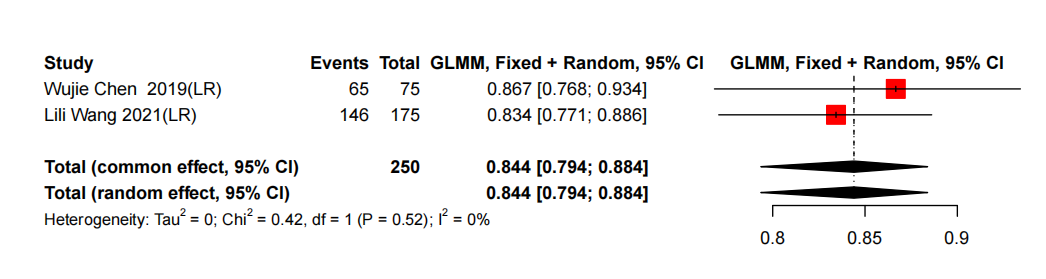


eFigure 20 The overall pooled accuracy for predicting lymph node metastasis of advanced gastric cancer in test set
